# Cardiometabolic effects of contrast therapy: A systematic review

**DOI:** 10.1113/EP093612

**Published:** 2026-08-29

**Authors:** Veerle de Haan, Guy Plasqui, Hannah Pallubinsky

**Affiliations:** ^1^ Department of Nutrition and Movement Sciences, Institute for Nutrition and Translational Research in Metabolism (NUTRIM), Faculty of Health, Medicine and Life Sciences Maastricht University Maastricht the Netherlands

**Keywords:** cardiometabolic health, cardiovascular health, cold therapy, contrast therapy, heat therapy, metabolic health, thermal therapy

## Abstract

The rising prevalence of overweight and obesity, together with associated cardiometabolic disorders, underscores the need for additional strategies alongside established interventions. The growing popularity of contrast therapy – alternating exposure to heat and cold, a practice common in traditional Finnish sauna bathing – has prompted interest in its potential cardiovascular and metabolic health benefits. This systematic review outlines the acute and chronic effects of contrast therapy on cardiometabolic outcomes. The review followed PRISMA guidelines and searched three databases (PubMed, Scopus, Web of Science) until 17 June 2025. Interventional studies reporting cardiovascular and/or metabolic outcomes were included; 21 studies were identified, of which 19 contributed to data synthesis. Continuous outcomes were converted to standardised mean differences to enable comparison. Little evidence for effects of contrast therapy was found on resting heart rate, blood pressure or vascular health, all indicators of cardiovascular risk, and metabolic outcomes, including glucose and lipid metabolism. Some evidence of heat adaptation was observed, indicated by attenuated acute heart rate and core temperature responses, and decreased resting core temperature upon contrasting thermal exposure. However, the evidence base is limited, restricting the ability to draw firm conclusions or provide clear recommendations. Well‐designed, controlled studies, with systematic assessment of vascular and metabolic outcomes, are needed to further clarify the acute and long‐term effects of contrast therapy on cardiometabolic health.

## Abstract (highlights)

- **What is the topic of this review?**
  This paper provides the first comprehensive systematic review of the cardiometabolic effects of contrast therapy.
- **What advances does it highlight?**
  Chronic contrast therapy induces a heat‐acclimatised phenotype, despite intermittent cold exposure. However, no consistent evidence was found for effects on metabolic or vascular health following chronic contrast therapy. High‐quality, randomised controlled trials are needed to clarify the acute and long‐term cardiometabolic effects of contrast therapy.

## 1 INTRODUCTION

The rising global prevalence of overweight and obesity has emerged as a major public health crisis. In 2022, the World Health Organization (WHO) estimated there are 2.5 billion adults living with overweight and 890 million adults living with obesity globally (WHO, 2025b). Overweight and obesity contribute to insulin resistance and are associated with chronic low‐grade inflammation, type 2 diabetes (T2D) and cardiovascular disease (CVD) (Goossens, 2008; Klein et al., 2022; Poirier et al., 2006). Given that CVD remains the leading cause of death worldwide, accounting for an estimated 19.8 million deaths in 2022 (WHO, 2025a), there is considerable interest in interventions that may prevent CVD and improve cardiometabolic health. Consequently, additional therapies – used alongside established strategies such as dietary management, behavioural change and pharmacological interventions – are increasingly being investigated.

Thermotherapy, defined as exposure to heat or cold without exercise, has gained increasing interest in recent decades for its potential health benefits (Ivanova & Blondin, 2021; Pizzey et al., 2021). Various modalities, including water‐perfused suits (Ganio et al., 2011; Sellers et al., 2024), climate‐controlled environments (Hanssen et al., 2015; Pallubinsky et al., 2020) and hot‐ or cold‐water immersion (HWI; CWI) (Brazaitis et al., 2014; James et al., 2023), have been investigated as alternative strategies for improving cardiometabolic health.

Passive heat therapy, as a singular thermal stimulus, has been extensively studied for its potential cardiovascular benefits and its application in at‐risk populations (Brunt & Minson, 2021; Pizzey et al., 2021; Rodrigues et al., 2025), with more recent work extending to metabolic outcomes (Laukkanen & Kunutsor, 2024; Maley et al., 2019; Sebok et al., 2021). Acute passive heat exposure produces cardiovascular and thermoregulatory adjustments that facilitate heat loss and maintain a stable core temperature, including increases in heart rate (HR), sweat rate, peripheral blood flow and cardiac output, alongside reductions in blood pressure (BP) and systemic vascular resistance (Atencio et al., 2025; Behzadi et al., 2022; Gravel et al., 2021; Leach et al., 2024; Schlader et al., 2019). Repeated heat exposure, however, attenuates this acute cardiovascular strain (Hoekstra et al., 2018; Leppaluoto et al., 1986), reduces resting BP (Brunt et al., 2016; Hoekstra et al., 2018; Pallubinsky et al., 2017), and improves endothelial function and arterial stiffness (Brunt et al., 2016). Furthermore, while acute heat exposure appears to impair glucose handling (Faure et al., 2016; Maley et al., 2023), repeated exposure is reported to improve glycaemic control and insulin sensitivity (Ely et al., 2019; Hesketh et al., 2019; Hoekstra et al., 2018; James et al., 2023; Pallubinsky et al., 2020).

Similarly, research on passive cold therapy has explored both cardiovascular and metabolic effects (Esperland et al., 2022; Ivanova & Blondin, 2021; Jdidi et al., 2024). Acute passive cold exposure elicits cardiovascular responses that promote heat conservation through vasoconstriction, characterised by increased BP, stroke volume, and a transient rise in HR followed by a decrease (Reed et al., 2023; Sellers et al., 2021; Zalewski et al., 2014). The biphasic HR response is consistent with the initial cold shock response followed by baroreceptor‐mediated suppression (Jdidi et al., 2024; Tipton et al., 2017). Decreases in core and skin temperature are also consistently noted (Blondin et al., 2015; Brazaitis et al., 2014; Brenner et al., 1999; Reed et al., 2023). In contrast, repeated exposure to cold reduces resting BP (Sellers et al., 2024; Versteeg et al., 2023) and attenuates acute HR and BP responses (Brazaitis et al., 2014; Jansky et al., 1996). Acute cold exposure can also lower fasting plasma glucose (FPG) and fasting plasma insulin (FPI) concentrations in individuals with T2D (Blondin et al., 2015). Repeated cold exposure appears to enhance glucose tolerance and insulin sensitivity in healthy individuals and those with overweight and/or T2D (Blondin et al., 2014; 2017; Hanssen et al., 2015; Sellers et al., 2024). Such positive effects on glucose homeostasis may be attenuated when cold‐induced shivering is prevented (Remie et al., 2021).

Contrast therapy involves alternating periods of both heat and cold exposure, with traditional Finnish sauna bathing being the most commonly used and widely studied form (Laukkanen & Kunutsor, 2024). Sauna bathing typically consists of repeated heating periods of 5–20 min, interspersed by brief cooling periods, such as CWI or cold showers (Heinonen & Laukkanen, 2018). Although primarily used for relaxation and recreation, sauna bathing has increasingly been associated with favourable health outcomes (Heinonen & Laukkanen, 2018; Laukkanen & Kunutsor, 2024). Large prospective cohort studies have reported inverse associations between sauna frequency and duration and the risk of sudden cardiac death, fatal coronary heart disease (CHD), CVD and all‐cause mortality (Laukkanen et al., 2015, 2018). Furthermore, in certain cultures, such as those of Finland, Norway and Sweden, sauna bathing is already embedded in regular life and health practices. This widespread acceptance and regular use, commonly practised one to several times per week (Laukkanen et al., 2018), suggests that sauna‐based contrast therapy may represent a feasible therapeutic approach with potential applicability across different geographical and cultural settings.

Although beneficial health effects of contrast therapy have been partly noted (Heinonen & Laukkanen, 2018; Laukkanen & Kunutsor, 2024), the literature remains heterogeneous and has not yet been systematically synthesised. Therefore, this review aimed to systematically evaluate the existing literature on the cardiovascular and metabolic effects of acute and chronic contrast therapy.

## 2 METHODS

The review followed the Preferred Reporting Items for Systematic Reviews and Meta‐Analyses (PRISMA) (2020) guidelines (Page et al., 2021).

### 2.1 Eligibility criteria

Studies were included if they complied with the following criteria: (i) experimental study design; (ii) full‐text availability; (iii) English publication or English translation availability; (iv) adult participants (≥18 years); (v) investigation of a passive contrast therapy protocol (combination of passive heating and cooling); and (vi) reporting of cardiovascular and/or metabolic outcomes.

Exclusion criteria were observational studies, reviews, meta‐analyses and abstracts, as well as studies involving active heating/cooling (e.g., exercise‐based protocols) or partial thermal exposure (e.g., single‐limb heating).

### 2.2 Information sources and search strategy

Systematic searches were conducted in PubMed, Scopus and Web of Science using keywords covering cold and heat exposure and specific outcome measures. An overview of the full search strategy is enclosed in Appendix A. Initial combined searches identified few eligible studies, as many omitted both intervention components in the title or abstract. Therefore, the search strategy was supplemented by cold‐ and heat‐only interventions. Eligibility regarding the intervention type was confirmed through abstract and full‐text screening. In addition, filters were applied to ensure English‐language availability and study designs consistent with the eligibility criteria. Additional studies were identified through citation tracking and reviewing works of key authors identified in the initial search.

### 2.3 Selection process

Search results were uploaded to the review platform Rayyan (Ouzzani et al., 2016), where duplicates were flagged and manually removed. Titles and abstracts were also screened using Rayyan. After initial screening, studies were evaluated based on study design, intervention and outcome measures. No additional information was sought or confirmed with the study investigators. The screening and study selection were performed by one author (V.H.). The PRISMA flowchart was used to document the selection process.

### 2.4 Data collection process

Data were manually extracted into Excel spreadsheets for descriptive information, results and risk‐of‐bias. Data extraction was performed by one author (V.H.). No additional information was sought or confirmed with the study investigators. When multiple reports referred to the same study, data were extracted from all reports, but only the earliest publication was included in the synthesis to avoid duplicate reporting.

Outcomes were reported as means with standard deviations. Few studies that reported medians with confidence intervals (CI) or ranges were recorded accordingly. When the standard error of the mean was reported instead of the standard deviation, the standard deviation was calculated (SD=SEM×n). Graphical data were digitised using PlotDigitizer (PlotDigitizer, 3.1.6, 2025) when necessary.

### 2.5 Data items

Data were collected on participant and study characteristics, study design, risk of bias across relevant domains and selected outcomes. Three outcome domains were considered for this review: cardiovascular, metabolic and thermoregulatory. Cardiovascular and metabolic outcomes were predefined inclusion domains, but no restrictions were applied to specific outcomes within these domains during study selection. Thermoregulatory outcomes were not part of the inclusion criteria but were considered relevant to contrast therapy.

For the selected outcomes, baseline and post‐exposure values were collected along with between‐group comparisons where applicable. In studies involving repeated exposures, baseline and post‐exposure values for the final exposure were also collected.

### 2.6 Study risk of bias assessment

Methodological quality was assessed using the Cochrane Risk of Bias Tool for randomised controlled trials (RCTs), including additional crossover‐specific considerations, and the ROBINS‐I V2 tool (Sterne et al., 2016) used for non‐randomised studies. These tools evaluate the following domains: randomisation process, period and carryover effects, confounding, intervention classification, participant selection, deviations from intended interventions, missing data, outcome measurement, and selective reporting.

Each domain was rated as one of low, moderate/unclear or high risk of bias, and an overall risk of bias was summed up per study. Overall risk was classified as one of low (all domains rated low risk), some concerns (at least one domain rated some concerns but no high‐risk ratings) or high (one or more domains rated high risk, or multiple domains rated some concerns). The intervention classification domain was applied only to two‐group clinical study designs, as it was not relevant to single‐group designs. No additional information was sought or confirmed with the study investigators. The risk‐of‐bias assessment was performed by one author (V.H.).

### 2.7 Data synthesis

All outcomes were measured on continuous scales and converted to standardised mean differences (SMDs) to facilitate comparison across studies. Acute effects were defined as baseline‐to‐post‐exposure or between‐group comparisons. Chronic effects were assessed as baseline‐to‐post‐intervention measurements or differences between the first and final exposure (difference in differences (DiD)). For DiD analyses, change scores were calculated from the reported means. Standard deviations of the change scores were estimated from the reported standard deviations, assuming a pre‐post correlation of r = 0.5, as no correlation coefficients were reported (Appendix B). SMDs and corresponding CIs were estimated for within‐subject pre‐post comparisons and chronic effects using established formulas (see Appendix B). Small‐sample bias was corrected using Hedges’ *g*.

For within‐subject pre–post comparisons, positive and negative SMDs indicate increases and decreases, respectively. For DiD comparisons, positive effect sizes indicate either a smaller increase from first to last exposure or a larger decrease during the final exposure relative to the first. Thus, negative effect sizes indicate either a larger increase from first to last exposure, or a smaller decrease during the final exposure relative to the first exposure. SMDs of 0.20, 0.50, and 0.80 or greater were respectively interpreted as small, medium and large (Rosenthal, 1996). Effects were considered statistically significant when the associated CI excluded the null value. In addition to statistically significant findings, trends or noteworthy patterns were reported when CIs approached significance or when consistent effects were observed despite not meeting the significance criterion.

Due to the low number of included studies and the varying number of studies reporting each outcome, no statistical synthesis methods were applied. Instead, findings were synthesised narratively with effect ranges presented in both SMDs and original measurement units, and supported by a results table. This approach does not account for differences in study population size.

Study characteristics, risk‐of‐bias assessments and individual study results are presented in three separate tables. These were also used to explore heterogeneity descriptively. For some studies, only part of the reported study design was relevant to this review. As such, the applicable section is underlined in the descriptive table. The results table includes both author‐reported values and calculated SMDs and is organised by intervention duration (acute/chronic) and outcome category (cardiovascular, metabolic blood biomarkers, thermoregulatory, inflammatory response markers). In addition, forest plots displaying SMDs and 95% CIs are presented for outcomes with a sufficient number of studies to allow meaningful synthesis and facilitate interpretation of the evidence. Studies are ordered according to risk‐of‐bias rating, with the lowest risk shown at the top of each plot.

## 3 RESULTS

### 3.1 Study selection

**FIGURE 1:**
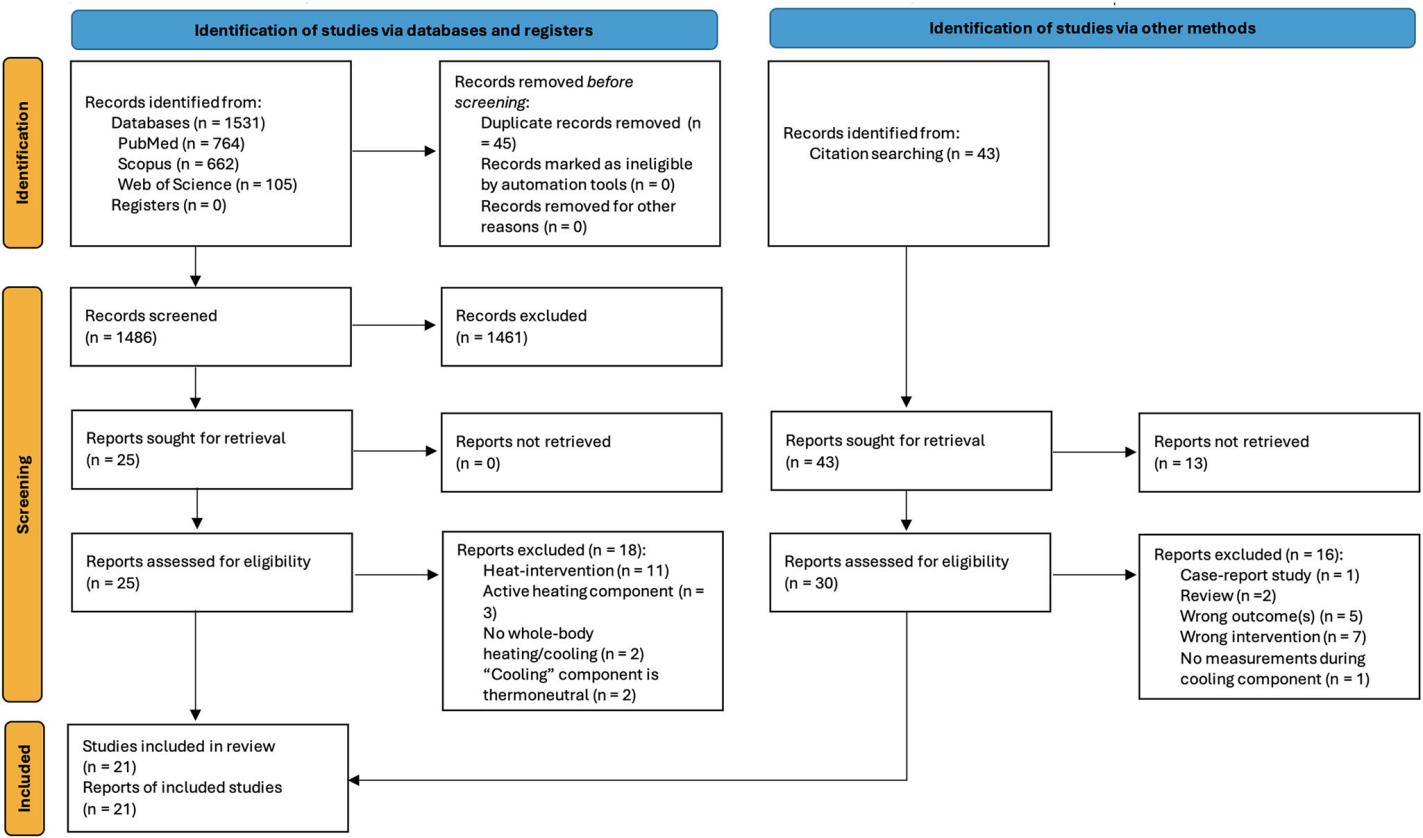
PRISMA 2020 flow diagram for new systematic reviews which included searches of databases, registers and other sources.

The study selection process is detailed in Figure 1. The initial database search (PubMed, Scopus, Web of Science) yielded 1531 records; 45 duplicates were removed, which left 1486 unique papers for screening. After title and abstract screening, 1461 papers were excluded, which left 25 articles for full‐text review. Of these, 18 were excluded because they did not meet the intervention criteria (i.e., heat‐only interventions, lack of whole‐body exposure, inclusion of an active heat component, or an insufficient cooling component).

An additional 43 papers were identified through cross‐referencing, of which 13 could not be retrieved due to language barriers or a lack of a digital record. Of the remaining 29 papers assessed in full, 16 were excluded for reasons such as incorrect publication type (e.g., case–control study or review), irrelevant outcomes or interventions, or absence of measurements taken during the cooling component. A total of 21 studies remained for inclusion in this systematic review.

### 3.2 Study characteristics

The included studies comprised one RCT, three crossover design studies, three two‐group clinical studies, and 14 single‐group clinical design studies, with several including subgroup analyses. The general characteristics of these studies are summarised in Table 1. Overall, the studies involved 853 participants (n = 710 for acute (83%); n = 143 for chronic (17%)), of whom 272 were women (32%). More than half of the included studies (n = 13) were conducted in Poland, while the remaining studies took place in Finland (n = 3), Canada (n = 2), Switzerland (n = 1), Slovakia (n = 1), and Russia (n = 1). Most studies had relatively small sample sizes, with 15 out of 21 involving 20 participants or fewer. The majority of the studies focused on younger healthy adults (n = 15) or sedentary adults (n = 1), but they also included cardiac patients (n = 3) and adults with cardiac risk factors (n = 2).

**TABLE 1:** General characteristics of the included studies.

Study ID	*n*	Population, country	Study design	Intervention	Final acute exposure heat or cold
Acute					
Dugué & Leppanen (2000)	20	Habitual and non‐habitual winter swimmers (19–64 years) 11F/9M, Finland	Single group clinical study with subgroup analysis	15‐min sauna (95°C; 30–50% RH) + 1‐min cold air (−15 to −5°C) + 30s CWI (ice‐cold outdoor) + 1‐min cold air	Cold
Gayda et al. (2012)	16	Untreated hypertensive patients (60.7 ± 9.4 years) 3F/13M, Canada	Randomised crossover design	(i) Resting control (C) period; (ii) 2 × 8‐min sauna (S) (85–90°C; 50–60% RH) ± 2‐min cold shower and 10‐min rest; (iii) 30‐min continuous exercise + 2 × 8‐min sauna and 2‐min cold shower	Heat
Jezova et al. (1994)	16	Healthy males and females (29–40 years) 8F/8M, Slovakia	Single group clinical study with subgroup analysis	20‐min sauna (80°C; 10% RH) + 30s CWI (18°C) + 30‐min covered in a blanket	Cold^$^
Kunutsor et al. (2018)^a^	102	Asymptomatic adults with >1 cardiovascular risk factor (51.9 ± 9.2 years) 46F/56M, Finland	Single group clinical study with subgroup analysis	2 × 15‐min sauna (73°C; 10–20% RH) + 2‐min shower	Heat
Lee et al. (2018)^a^	102	Asymptomatic adults with >1 cardiovascular risk factor (51.9 ± 9.2 years) 46F/56M, Finland	Single group clinical study	2 × 15‐min sauna (73 ± 2°C; 10–20% RH) + 2‐min shower	Heat
Pilch et al. (2005)	20	Swimmers (T) vs. non‐training (U) students (20–23 years) 0F/20M, Poland	Single group clinical study with subgroup analysis	3 × 15‐min sauna (92.3°C; 27.4% RH) + 2‐min rest (20–22°C shower)	Heat
Pilch et al. (2013)	18	Male athletes (A) and non‐athletes (N) (21.6 ± 0.52 years; 21.7 ± 0.48 years) 0F/18M, Poland	Single group clinical study with subgroup analysis	15‐min sauna (96 ± 2°C; 15 ± 3% RH) + 2‐min cool‐down (shower, 19–20°C). Repeated until *T*_re_ +1.2°C	Cold
Pilch et al. (2014)	10	Healthy males (25–28 years) 0F/10M, Poland	Non‐randomised crossover design	3 × 15‐min sauna + 5‐min break (incl. 2‐min cooling, 22°C water). I = dry (D) (91°C; 5–18% RH); II = wet (W) (59°C; 60.5% RH)	Heat
Podstawski et al. (2019)	45	Male students (19–24 years) 0F/45M, Poland	Single group clinical study	4 × 10‐min sauna (90°C; 14–16% RH) + 5‐min recovery (18°C room; 14–15°C shower + 10°C paddling pool)	Cold
Podstawski et al. (2020)	55	Sedentary university students (18–23 years) 0F/55M, Poland	Two group, non‐randomised clinical study	16‐min sauna (90–92°C; 14–16% RH) + (I) 2‐min paddling pool (C) (10–11°C) or (II) 30s shower (S) (10–11°C) and 90s rest (20–22°C; ∼50% RH)	Cold
Podstawski et al. (2021)	30	Male volunteers (19–26 years) 0F/30M, Poland	Single group clinical study	4 × 12‐min sauna (90°C; 14–16% RH) + 6‐min breaks (incl. 1‐min cold paddling pool (10–11°C))	Cold
Prystupa et al. (2009)	173	Healthy students (20.99 ± 0.67 years F; 21.42 ± 1.30 years M) 100F/73M, Poland	Single group clinical study with subgroup analysis	3×: 12‐min sauna (115°C; 35% RH) + 3 CWIs (10°C) + 24‐min rest phase	Cold
Radtke et al. (2016)	37	CHF (*n* = 12; 61.8 ± 9.2 years) and CAD patients (*n* = 13; 61.2 ± 10.6 years), and control subjects (*n* = 12; 60.9 ± 8.9 years) 0F/37M, Switzerland	Single group clinical study with subgroup analysis	2 × 10‐min sauna (80°C; 36 ± 1% RH) + short shower (moderately cold temperature) + CWI (12°C) for a few seconds.	Cold
Sudakov et al. (1988)	6	Healthy males (28–50 years) 0F/6M, Russia	Non‐randomised crossover design	6×: sauna (80–85°C) until discomfort (9.9 ± 1.5 min) + (i) 5–6 min of rest at room temperature (22–26°C), or (ii) cool shower (16–18°C) + contrast‐cooling pool (18–22°C) for 30–60s	Cold
Chronic					
Debray et al. (2023)	41	Adults with stable CAD (62 ± 6 years C; 63 ± 6 years S) 8F/33M, Canada	RCT	8 weeks (4 days/week) of (i) 2 × 10‐15 min sauna (79 ± 3°C; 13 ± 4% RH) + 5‐min rest (incl. 30s cold shower); (ii) control	Heat
Gryka et al. (2014)^*^	16	Physically active, non‐smoker males (20–23 years) 0F/16M, Poland	Single group clinical study	10 sessions (every one or two days) of 3 × 15‐min sauna (90 ± 2°C; 5–16% RH) + 2‐min cooling (cold water stream, 20°C)	Cold
Gryka et al. (2020)^b^ ^*^	20	Healthy trained (T) and untrained (U) males (20–25 years) 0F/20M, Poland	Single group clinical study with subgroup analysis	10 sessions of 3 × 15‐min sauna (90 ± 2°C; 5–16% RH) with 2 min cooling breaks (running water, ∼20°C)	Cold
Pilch et al. (2007)^c^, ^*^	20	Healthy students (19–21 years) 20F/0M, Poland	Two group, non‐randomised clinical study	7 sessions (every second day) of (i) 30‐min sauna (80°C; 5–27% RH); (ii) 45‐min sauna incl. 5‐min break (2‐min shower, 20–22°C; 3‐min rest)	Heat
Pilch et al. (2008)^c^, ^*^	10	Healthy females (19–21 years) 10F/0M, Poland	Single group clinical study	7 sessions (every second day) of 45‐min sauna (80.1°C; 5–26.6% RH) incl. 5‐min break (shower, ∼20–22°C)	Heat
Pilch et al. (2010)^c^, ^*^	20	Healthy non‐smoker females (19–21 years) 20F/0M, Poland	Two group, non‐randomised clinical study	7 sessions (every second day) of: (i) 30‐min sauna (80.1°C; 5–26.6% RH); (ii) 45‐min sauna incl. 5‐min break (2‐min shower, 20–22°C)	Heat
Pilch et al. (2023)^b^ ^*^	20	Healthy trained (T) and untrained (U) males (20–25 years) 0F/20M, Poland	Single group clinical study with subgroup analysis	10 exposures of 3 × 15‐min sauna (90 ± 2°C; 5–16% RH) + 2‐min breaks (running water, ∼20°C)	Heat

Abbreviations: CAD, coronary artery disease; CHF, chronic heart failure; CWI, cold‐water immersion; RCT, randomised controlled trial; RH, relative humidity; T_re_, rectal temperature.

* The asterisk indicates that the study has a chronic intervention but also includes acute analyses.

^$^ In this study, the final exposure was cooling; however, post‐exposure measurements were obtained after the preceding heating phase.

^a,b,c^ Papers derived from the same study.

Seventeen studies examined acute contrast interventions or conducted acute analyses within chronic protocols (Table 1, Acute). These interventions involved one to six successive sauna exposures lasting 8–20 min, interspersed with cooling phases of a few seconds to 2 min. Eight studies focused on chronic contrast interventions (Table 1, Chronic), comprising 7–32 sessions with sauna exposures of 10–20 min and cooling periods lasting 30 s to 5 min. Dry Finnish sauna was used as the heating component in 20 out of 21 studies with an aimed temperature range between 80 and 90°C and relative humidity (RH) levels of 10–50%. Only one study (Gayda et al., 2012) employed higher humidity levels (50%–60%) at high temperatures (85–90°C). Another study (Gryka et al., 2014) compared dry (91°C; 5–18% RH) and wet sauna (59°C; 60.5% RH).

Several included papers originated from the same study: Pilch et al. (2007, 2008, 2010); Lee et al. (2018) and Kunutsor et al. (2018); as well as Gryka et al. (2020) and Pilch et al. (2023). Data from Pilch et al. (2008) were excluded from the synthesis, as the paper did not report any new relevant findings. In addition, the study by Sudakov et al. (1988) was excluded from the data synthesis due to insufficient reporting of intervention design and data analysis, as well as inadequate outcome presentation, specifically the absence of statistical distributions. However, to provide a comprehensive overview of the current literature, both studies were retained in the general characteristics and risk of bias assessment tables.

### 3.3 Risk of bias in studies

Overall, most studies showed a high risk of bias, with some rated as low, unclear, or low risk with concerns regarding uncontrolled confounding. The quality assessment of the included studies is summarised in Table 2.

**TABLE 2:** Risk of bias assessment.

	Randomisation process^a^, ^b^	Period and carryover effects^b^	Confounding^c^	Intervention classification^c^		Participant selection^c^	Deviations from intended interventions	Missing data	Outcome measurement	Selective reporting	Overall
Debray et al. (2023)	++	N/A	N/A	N/A	N/A		++	++	++	++	++
Dugué & Léppanen (2000)	N/A	N/A	?	N/A	++		++	−	++	++	−
Gayda et al. (2012)	?	++	N/A	N/A	N/A		++	++	++	++	?
Gryka et al. (2014)	N/A	N/A	?	N/A	++		++	−	++	++	−
Gryka et al. (2020)	N/A	N/A	?	N/A	++		++	++	++	++	+
Jezova et al. (1994)	N/A	N/A	?	N/A	++		++	++	++	++	+
Kunutsor et al. (2018)	N/A	N/A	?	N/A	++		++	++	++	++	+
Lee et al. (2018)	N/A	N/A	?	N/A	++		++	++	++	++	+
Pilch et al. (2005)	N/A	N/A	−	N/A	++		++	−	++	++	−
Pilch et al. (2007)	N/A	N/A	?	−	?		++	−	++	++	−
Pilch et al. (2008)	N/A	N/A	++	N/A	++		++	−	++	++	−
Pilch et al. (2010)	N/A	N/A	?	−	?		++	−	++	++	−
Pilch et al. (2013)	N/A	N/A	?	N/A	++		++	−	++	++	−
Pilch et al. (2014)	?	++	N/A	N/A	N/A		++	?	−	++	−
Pilch et al. (2023)	N/A	N/A	++	N/A	++		++	−	++	++	−
Podstawski et al. (2019)	N/A	N/A	?	N/A	++		++	++	++	++	+
Podstawski et al. (2020)	N/A	N/A	?	−	?		++	++	++	++	−
Podstawski et al. (2021)	N/A	N/A	++	N/A	++		++	++	++	++	++
Prystupa et al. (2009)	N/A	N/A	−	N/A	++		?	?	++	?	−
Radtke et al. (2016)	N/A	N/A	?	N/A	++		++	++	++	++	+
Sudakov et al. (1988)	?	−	N/A	N/A	N/A		?	++	++	−	−

++: low risk of bias; +: low risk of bias with concerns about uncontrolled confounding; ?: unclear risk of bias; −: high risk of bias.

^a^ Applicable to randomised controlled trials assessed with the Cochrane Risk of Bias tool.

^b^ Applicable to cross‐over trials assessed with the Cochrane Risk of Bias tool.

^c^ Applicable to non‐randomised trials assessed with the ROBINS‐I tool.

Randomisation was clearly reported in the RCT (Debray et al., 2023), but was incomplete or absent in the crossover trials (Gayda et al., 2012; Pilch et al., 2014; Sudakov et al., 1988). One crossover study lacked a washout period, posing a high risk of carryover effects (Sudakov et al., 1988). The same study provided insufficient information on possible deviations from intended interventions, resulting in an unclear risk (Sudakov et al., 1988). Pilch et al. (2014) had an unclear risk for missing data due to unreported sample sizes alongside the results. The same study was rated high for outcome measurement bias because HR was assessed via carotid palpation, a method prone to inter‐rater variability. Selective reporting bias was high in one study (Sudakov et al., 1988), as no analysis plan was reported, making the conducted analyses unclear.

Among the clinical studies, the risk of confounding was unclear in 11 studies and high in two (Table 2), with limited control of confounders. The three two‐group studies (Pilch et al., 2010, 2007; Podstawski et al., 2020) had a high risk of intervention classification and unclear participant selection, as allocation procedures were not described. Prystupa et al. (2009) showed an unclear risk of deviations from intended interventions due to insufficient statistical detail. Missing data posed a high risk in seven studies and unclear risk in one (Table 2) due to inconsistencies in reported sample sizes. All studies were low risk for outcome measurement, with consistent methods across participants. Selective reporting was unclear in one study (Prystupa et al., 2009) that lacked a statistical analysis section.

### 3.4 Acute effects of contrast therapy

The acute effects of contrast therapy are summarised in Table 3. Several studies required data transformation to enable effect size calculation. SDs were derived from SEMs reported by Jezova et al. (1994) and Radtke et al. (2016). None of the included studies reported SMDs, which were therefore calculated (see Methods, Data synthesis, Appendix B). SMDs could not be computed for Kunutsor et al. (2018) and Dugué & Leppanen (2000), as results were reported as medians with 95% CIs and means with minimal and maximal values, respectively. Furthermore, Pilch et al. (2005) did not report sufficient measures of statistical distribution to permit SMD calculation.

**TABLE 3:** Study outcomes and effect sizes.

Outcome	Study ID	RoB	Comparison (*n*)	Results (mean ± SD (sig.))	SMD [95%CI]	Index
Acute						
Cardiovascular						
AIx	Lee et al. (2018)^a^, ^$^	+	Pre− vs. post (102)	9.8 ± 16.0 vs. 4.1 ± 15.8 (*P* < 0.001)	−0.355 [−0.630; −0.081]	↓^*^
			Pre‐ vs. 30‐min post (102)	9.8 ± 16.0 vs. 9.0 ± 1.7 (NS)	−0.053 [−0.325; 0.220]	↔
AP [mmHg]	Lee et al. (2018)^a^, ^$^	+	Pre‐ vs. post (102)	1.5 ± 0.9 vs. 1.6 ± 0.8 (NS)	0.116 [−0.157; 0.389]	↔
			Pre‐ vs. 30‐min post (102)	1.5 ± 0.9 vs. 1.4 ± 0.9 (NS)	−0.110 [−0.383; 0.163]	↔
CO [L min^−1^]	Gayda et al. (2012)	?	Pre‐ vs. post (16)^#^	5.3 ± 1.5 vs. 7.4 ± 1.8 (*P* < 0.0001)	1.197 [0.461; 1.933]	↑↑↑^*^
			Pre‐ vs. 15‐min post (16)	5.3 ± 1.5 vs. 5.2 ± 1.5 (NS)	−0.064 [−0.724; 0.596]	↔
			Pre‐ vs. 120‐min post (16)	5.3 ± 1.5 vs. 5.2 ± 1.2 (NS)	−0.070 [−0.730; 0.590]	↔
			C vs. S baseline (16)	5.0 ± 1.1 vs. 5.3 ± 1.5 (NS)	0.212 [−0.450; 0.875]	↑
			C vs. S 15‐min post (16)	5.0 ± 1.3 vs. 5.2 ± 1.5 (*P* < 0.05)	0.135 [−0.526; 0.796]	↔
			C vs. S 120‐min post (16)	5.0 ± 1.2 vs. 5.2 ± 1.2 (*P* < 0.05)	0.159 [−0.502; 0.820]	↔
	Radtke et al. (2016)^#^	+	Pre‐ vs. post (CHF) (12)	4.42 ± 0.756 vs. 6.25 ± 1.432 (?)	1.381 [0.395; 2.367]	↑↑↑^*^
			Pre‐ vs. post (CAD) (13)	4.75 ± 0.985 vs. 6.59 ± 1.102 (?)	1.652 [0.785; 2.519]	↑↑↑^*^
			Pre‐ vs. post (C) (12)	5.33 ± 1.49 vs. 6.81 ± 1.47 (?)	0.936 [0.142; 1.730]	↑↑↑^*^
DBP [mmHg]	Gayda et al. (2012)	?	Pre‐ vs. post (16)^#^	86 ± 8; 82 ± 5 (NS)	−0.544 [−1.214; 0.126]	↓↓
			Pre‐ vs. 15‐min post (16)	86 ± 8 vs. 85 ± 18 (NS)	−0.061 [−0.722; 0.600]	↔
			Pre‐ vs. 120‐min post (16)	86 ± 8 vs. 84 ± 8 (NS)	−0.238 [−0.901; 0.425]	↓
			C vs. S baseline (16)	85 ± 8 vs. 86 ± 8 (NS)	0.119 [−0.542; 0.780]	↔
			C vs. S 15‐min post (16)	83 ± 10 vs. 85 ± 18 (NS)	0.122 [−0.540; 0.784]	↔
			C vs. S 120‐min post (16)	84 ± 7 vs. 84 ± 8 (NS)	0.000 [−0.660; 0.660]	↔
	Gryka et al. (2020)^b^	+	Pre‐ vs. post (T) (10)	80.0 ± 4.1 vs. 71.0 ± 7.75 (*P* < 0.01)	−1.237 [−2.262; −0.213]	↓↓↓^*^
			Pre‐ vs. post (U) (10)	79.5 ± 4.4 vs. 69.5 ± 6.85 (*P* < 0.01)	−1.535 [−2.574; −0.498]	↓↓↓^*^
	Lee et al. (2018)^a^, ^$^	+	Pre‐ vs. post (102)	82.1 ± 9.6 vs. 75.1 ± 9.3 (*P* < 0.001)	−0.735 [−1.016; −0.454]	↓↓^*^
			Pre‐ vs. 30‐min post (102)	82.1 ± 9.6 vs. 80.6 ± 9.2 (NS)	−0.158 [−0.431; 0.114]	↔
	Pilch et al. (2007)^c^, ^$^	−	Pre‐ vs. post (10)	72 ± 7.8 vs. 52 ± 18.6 (*P* < 0.01)	−1.141 [−2.233; −0.049]	↓↓↓^*^
	Pilch et al. (2014)^$^	−	Pre‐ vs. post (W) (10)	77.7 ± 4.76 vs. 57.7 ± 4.16 (*P* < 0.01)	−4.111 [−5.561; −2.663]	↓↓↓^*^
			Pre‐ vs. post (D) (10)	78.7 ± 5.72 vs. 63.7 ± 5.29 (*P* < 0.01)	−2.510 [−3.613; −1.406]	↓↓↓^*^
	Podstawski et al. (2019)	+	Pre‐ vs. post 4 (45)^§^	81.38 ± 5.97 vs. 94.27 ± 9.00 (?)	1.598 [1.086; 2.109]	↑↑↑^*^
	Podstawski et al. (2020)	−	Pre‐ vs. post (C) (30)^§^	77.8 ± 11.4 vs. 69.9 ± 8.7 (?)	−0.747 [−1.255; −0.239]	↓↓^*^
			Pre‐ vs. post (S) (25)^§^	78.6 ± 8.0 vs. 76.3 ± 8.4 (?)	−0.272 [−0.812; 0.269]	↓
	Podstawski et al. (2021)	++	Pre‐ vs. post (30)	78.20 ± 12.05; 71.27 ± 9.76 (*P* < 0.001)	−0.272 [−0.812; 0.269]	↓
	Prystupa et al. (2009)	−	Pre‐ vs. post 3 (F) (100)	80.82 ± 12.48 vs. 82.92 ± 16.77 (*P =* 0.1197)	−1.067 [−1.361; −0.774]	↓↓↓^*^
			Pre‐ vs. post 3 (M) (73)	72.96 ± 13.42 vs. 75.73 ± 15.94 (*P =* 0.0377)	−0.895 [−1.232; −0.557]	↓↓↓^*^
	Radtke et al. (2016)^#^	+	Pre‐ vs. post (CHF) (12)^§^	106.46 ± 12.94 vs. 107.78 ± 10.28 (?)	0.105 [−0.645; 0.854]	↔
			Pre‐ vs. post (CAD) (13)^§^	111.39 ± 8.63 vs. 112.98 ± 9.68 (?)	0.163 [−0.563; 0.888]	↔
			Pre‐ vs. post (C) (12)^§^	122.07 ± 10.87 vs. 120.47 ± 11.85 (?)	−0.131 [−0.882; 0.619]	↔
DT [ms]	Lee et al. (2018)^a^, ^$^	+	Pre‐ vs. post (102)	635.1 ± 115.1 vs. 494.6 ± 113.0 (*P* < 0.001)	−1.223 [−1.520; −0.926]	↓↓↓^*^
			Pre‐ vs. 30‐min post (102)	635.1 ± 115.1 vs. 634.3 ± 121.2 (NS)	−0.007 [−0.279; 0.266]	↔
EDV [mL]	Gayda et al. (2012)	?	Pre‐ vs. post (16)^#^	162 ± 46; 161 ± 45 (NS)	−0.021 [−0.681; 0.639]	↔
			Pre‐ vs. 15‐min post (16)	162 ± 46 vs. 162 ± 46 (NS)	0.000 [−0.660; 0.660]	↔
			Pre‐ vs. 120‐min post (16)	162 ± 46 vs. 176 ± 43 (*P* < 0.0001)	0.299 [−0.365; 0.963]	↑
			C vs. S baseline (16)	163 ± 46 vs. 162 ± 46 (NS)	−0.021 [−0.681; 0.639]	↔
			C vs. S 15‐min post (16)	168 ± 51 vs. 162 ± 46 (NS)	−0.117 [0.778; 0.543]	↔
			C vs. S 120‐min post (16)	174 ± 52 vs. 176 ± 43 (NS)	0.040 [−0.620; 0.700]	↔
Hct [vol. fraction]	Dugué & Léppanen (2000)	−	Pre‐ vs. post (20)	40 (34; 45) vs. 42 (36; 47) (*P* < 0.001)	N/A	N/A
Hct [%]	Podstawski et al. (2021)	++	Pre‐ vs. post (30)	44.09 ± 2.45 vs. 44.59 ± 3.15 (NS)	−0.171 [−0.325; 0.665]	↔
HR [bpm]	Gayda et al. (2012)	?	Pre‐ vs. post (16)^#^	75 ± 11; 99 ± 12 (*P* < 0.0001)	1.982 [1.149; 2.815]	↑↑↑^*^
			Pre‐ vs. 15‐min post (16)	75 ± 11 vs. 77 ± 10 (*P* < 0.0001)	0.181 [−0.481; 0.842]	↔
			Pre‐ vs. 120‐min post (16)	75 ± 11 vs. 71 ± 10 (NS)	−0.362 [−1.027; 0.304]	↓
			C vs. S baseline (16)	72 ± 10 vs. 75 ± 11 (NS)	0.271 [−0.392; 0.935]	↑
			C vs. S 15‐min post (16)	71 ± 11 vs. 77 ± 10 (*P* < 0.0001)	0.542 [−0.130; 1.214]	↑↑
			C vs. S 120‐min post (16)	70 ± 11 vs. 71 ± 10 (*P* < 0.0001)	0.090 [−0.570; 0.750]	↔
	Gryka et al. (2014)^$^	−	Pre‐ vs. post (16)	69.00 ± 5.37 vs. 132.00 ± 6.53 (*P =* 0.0004)	9.943 [7.128; 12.759]	↑↑↑^*^
	Gryka et al. (2020)^b^	+	Pre‐ vs. post (T) (10)	67.6 ± 6.1 vs. 131.6 ± 5.5 (*P* < 0.01)	10.145 [7.037; 13.254]	↑↑↑^*^
			Pre‐ vs. post (U) (10)	74.0 ± 6.9 vs. 136.4 ± 9.7 (*P* < 0.01)	6.661 [3.951; 9.372]	↑↑↑^*^
	Jezova et al. (1994)^#^	+	Pre‐ vs. post (F) (8)	71.23 ± 7.37 vs. 142.64 ± 10.63 (*P* < 0.01)	6.838 [3.689; 9.997]	↑↑↑^*^
			Pre‐ vs. 15‐min post (F) (8)	71.23 ± 7.37 vs. 73.24 ± 4.47 (NS)	0.283 [−0.607; 1.171]	↑
			Pre‐ vs. 30‐min post (F) (8)	71.23 ± 7.37 vs. 70.45 ± 4.82 (NS)	−0.108 [−0.994; 0.777]	↔
			Pre‐ vs. post (M) (8)	71.87 ± 9.28 vs.121.48 ± 9.84 (*P* < 0.01)	4.681 [2.788; 6.574]	↑↑↑^*^
			Pre‐ vs. 15‐min post (M) (8)	71.87 ± 9.28 vs. 79.53 ± 3.68 (NS)	0.854 [−0.067; 1.777]	↑↑↑
			Pre‐ vs. 30‐min post (M) (8)	71.87 ± 9.28 vs. 72.05 ± 3.07 (NS)	0.020 [−0.865; 0.905]	↔
	Lee et al. (2018)^a^, ^$^	+	Pre‐ vs. post (102)	65.2 ± 10.4 vs. 80.7 ± 15.1 (*P* < 0.001)	1.149 [0.842; 1.457]	↑↑↑^*^
			Pre‐ vs. 30‐min post (102)	65.2 ± 10.4 vs. 65.9 ± 10.7 (NS)	0.066 [−0.206; 0.338]	↔
	Pilch et al. (2005)^$^	−	Pre‐ vs. post (T) (10)	74.2 ± ? vs. 134.1 ± ? (*P =* 0.01)	N/A	N/A
			Pre‐ vs. post (U) (10)	78.9 ± ? vs. 141.9 ± ? (?)	N/A	N/A
	Pilch et al. (2007)^c^, ^$^	−	Pre‐ vs. post (10)	67 ± 10.55 vs. 118 ± 18.13 (*P* < 0.005)	2.985 [1.383; 4.587]	↑↑↑^*^
	Pilch et al. (2014)^$^	−	Pre‐ vs. post (W) (10)	66.2 ± 2.28 vs. 138.2 ± 1.99 (*P* < 0.01)	30.917 [21.846; 39.986]	↑↑↑^*^
			Pre‐ vs. post (D) (10)	66.6 ± 2.84 vs. 126 ± 2.49 (*P* < 0.01)	20.442 [14.405; 26.480]	↑↑↑^*^
	Podstawski et al. (2019)	+	Pre‐ vs. post 4 (45)^§^	98.00 ± 7.41 vs. 133.36 ± 5.54 (?)	5.209 [4.412; 6.006]	↑↑↑^*^
	Podstawski et al. (2020)	−	Pre‐ vs. average (C) (30)^§^	82.1 ± 10.7 vs. 104.1 ± 8.5 (?)	2.191 [1.581; 2.801]	↑↑↑^*^
			Pre‐ vs. post (C) (30)^§^	82.1 ± 10.7 vs. 57.5 ± 6.8 (?)	−2.557 [−3.192; −1.921]	↓↓↓^*^
			Pre‐ vs. average (S) (25)^§^	80.2 ± 8.1 vs. 109.5 ± 8.3 (?)	3.464 [2.591; 4.336]	↑↑↑^*^
			Pre‐ vs. post (S) (25)^§^	80.2 ± 8.1 vs. 90.7 ± 12.7 (?)	0.914 [0.323; 1.506]	↑↑↑^*^
	Podstawski et al. (2021)	++	Pre‐ vs. post (30)	74.57 ± 12.99 vs. 82.00 ± 13.59 (*P =* 0.003)	0.545 [0.041; 1.048]	↑↑^*^
	Prystupa et al. (2009)	−	Pre‐ vs. post 3 (F) (100)	80.82 ± 12.48 vs. 82.92 ± 16.77 (*P =* 0.1197)	0.138 [−0.138; 0.414]	↔
			Pre‐ vs. post 3 (M) (73)	72.96 ± 13.42 vs. 75.73 ± 15.94 (*P =* 0.0377)	0.185 [−0.138; 0.507]	↔
	Radtke et al. (2016)^#^	+	Pre‐ vs. post (CHF) (12)^§^	73.07 ± 13.49 vs. 90.45 ± 17.14 (?)	1.040 [0.217; 1.864]	↑↑↑^*^
			Pre‐ vs. post (CAD) (13)^§^	68.54 ± 6.92 vs. 84.21 ± 11.37 (?)	1.486 [0.559; 2.413]	↑↑↑^*^
			Pre‐ vs. post (C) (12)^§^	69.35 ± 9.07 vs. 82.55 ± 11.28 (?)	1.194 [0.351; 2.036]	↑↑↑^*^
(L)VET [ms]	Gayda et al. (2012)	?	Pre‐ vs. post (16)^#^	330 ± 51; 290 ± 44 (*P* < 0.0001)	−0.795 [−1.480; −0.110]	↓↓^*^
			Pre‐ vs. 15‐min post (16)	330 ± 51 vs. 302 ± 61 (*P* < 0.0001)	−0.470 [−1.143; 0.202]	↓
			Pre‐ vs. 120‐min post (16)	330 ± 51 vs. 327 ± 50 (*P* < 0.0001)	−0.056 [−0.717; 0.604]	↔
			C vs. S baseline (16)	325 ± 38 vs. 330 ± 51 (NS)	0.104 [−0.557; 0.765]	↔
			C vs. S 15‐min post (16)	325 ± 56 vs. 302 ± 61(NS)	−0.373 [−1.040; 0.293]	↓
			C vs. S 120‐min post (16)	306 ± 60 vs. 327 ± 50 (*P* < 0.001)	0.359 [−0.306; 1.024]	↑
	Lee et al. (2018)^a^, ^$^	+	Pre‐ vs. post (102)	307.4 ± 26.4 vs. 277.9 ± 37.8 (*P* < 0.001)	−0.872 [−1.164; −0.579]	↓↓↓^*^
			Pre‐ vs. 30‐min post (102)	307.4 ± 26.4 vs. 299.0 ± 34.1 (*P* < 0.001)	−0.269 [−0.543; 0.005]	↓
MAP [mmHg]	Lee et al. (2018)^a^, ^$^	+	Pre‐ vs. post (102)	99.4 ± 15.0 vs. 93.6 ± 10.3 (*P* < 0.001)	−0.433 [−0.708; −0.158]	↓^*^
			Pre‐ vs. 30‐min post (102)	99.4 ± 15.0 vs. 95.9 ± 14.2 (NS)	−0.237 [−0.511; 0.036]	↓
PP [mmHg]	Lee et al. (2018)^a^, ^$^	+	Pre‐ vs. post (102)	42.7 ± 9.2 vs. 44.9 ± 9.8 (NS)	0.229 [−0.044; 0.503]	↑
			Pre‐ vs. 30‐min post (102)	42.7 ± 9.2 vs. 39.3 ± 8.3 (*P* < 0.001)	−0.384 [−0.659; −0.109]	↓^*^
PPa [%]	Lee et al. (2018)^a^, ^$^	+	Pre‐ vs. post (102)	28.8 ± 10.1 vs. 25.9 ± 13.4 (NS)	−0.238 [−0.512; 0.036]	↓
			Pre‐ vs. 30‐min post (102)	28.8 ± 10.1 vs. 27.5 ± 10.8 (NS)	−0.123 [−0.396; 0.150]	↔
PWV [m s^−1^]	Lee et al. (2018)^a^, ^$^	+	Pre‐ vs. post (102)	9.8 ± 2.4 vs. 8.6 ± 1.6 (*P* < 0.001)	−0.563 [−0.840; −0.286]	↓↓^*^
			Pre‐ vs. 30‐min post (102)	9.8 ± 2.4 vs. 9.0 ± 1.7 (NS)	−0.371 [−0.646; −0.097]	↓^*^
SBP [mmHg]	Gayda et al. (2012)	?	Pre‐ vs. post (16)^#^	136 ± 15; 139 ± 21 (NS)	0.152 [−0.510; 0.814]	↔
			Pre‐ vs. 15‐min post (16)	136 ± 15 vs. 136 ± 14 (NS)	0.000 [−0.660; 0.660]	↔
			Pre‐ vs. 120‐min post (16)	136 ± 15 vs. 139 ± 13 (NS)	0.203 [−0.459; 0.864]	↑
			C vs. S baseline (16)	134 ± 12 vs. 136 ± 15 (NS)	0.138 [−0.523; 0.800]	↔
			C vs. S 15‐min post (16)	135 ± 16 vs. 136 ± 14 (NS)	0.063 [−0.597; 0.723]	↔
			C vs. S 120‐min post (16)	142 ± 14 vs. 139 ± 13 (*P* < 0.05)	−0.211 [−0.874; 0.450]	↓
	Gryka et al. (2020)^b^	+	Pre‐ vs. post (T) (10)	124.0 ± 9.4 vs. 136.0 ± 8.8 (*P* < 0.01)	1.216 [0.328; 2.103]	↑↑↑^*^
			Pre‐ vs. post (U) (10)	116.5 ± 6.7 vs. 131.5 ± 8.2 (*P* < 0.01)	1.830 [0.799; 2.862]	↑↑↑^*^
	Lee et al. (2018)^a^, ^$^	+	Pre‐ vs. post (102)	136.5 ± 16.2 vs. 130.3 ± 14.4 (*P* < 0.001)	−0.400 [−0.675; −0.125]	↓^*^
			Pre‐ vs. 30‐min post (102)	136.5 ± 16.2 vs. 129.8 ± 13.8 (*P* < 0.001)	−0.439 [−0.715; −0.164]	↓^*^
	Pilch et al. (2007)^c^, ^$^	−	Pre‐ vs. post (10)	115 ± 9.8 vs. 128 ± 6.3 (*P* < 0.05)	1.396 [0.501; 2.288]	↑↑↑^*^
	Pilch et al. (2014)^$^	−	Pre‐ vs. post (W) (10)	123.4 ± 7.78 vs. 141.1 ± 8.56 (*P* < 0.01)	1.993 [0.954; 3.031]	↑↑↑^*^
			Pre‐ vs. post (D) (10)	122.6 ± 6.79 vs. 142.6 ± 4.96 (*P* < 0.01)	3.034 [1.866; 4.202]	↑↑↑^*^
	Podstawski et al. (2019)	+	Pre‐ vs. post 4 (45)^§^	125.84 ± 9.47 vs. 140.67 ± 7.55 (?)	1.681 [1.217; 2.146]	↑↑↑^*^
	Podstawski et al. (2020)	−	Pre‐ vs. post (C) (30)^§^	130.5 ± 9.2 vs. 122.0 ± 7.3 (?)	−0.985 [−1.504; −0.466]	↓↓↓^*^
			Pre‐ vs. post (S) (25)^§^	131.0 ± 9.6 vs. 127.3 ± 7.8 (?)	−0.406 [−0.948; 0.137]	↓
	Podstawski et al. (2021)	++	Pre‐ vs. post (30)	134.63 ± 12.95 vs. 124.93 ± 12.04 (*P* < 0.001)	−0.755 [−1.265; −0.245]	↓↓^*^
	Prystupa et al. (2009)	−	Pre‐ vs. post 3 (F) (100)	125.72 ± 16.01 vs. 122.88 ± 13.76 (*P =* 0.0565)	−0.188 [−0.463; 0.088]	↔
			Pre‐ vs. post 3 (M) (73)	147.56 ± 13.68 vs. 139.09 ± 13.20 (*P =* 0.0000)	−0.624 [−0.952; −0.295]	↓↓^*^
	Radtke et al. (2016)^#^	+	Pre‐ vs. post (CHF) (12)^§^	101.87 ± 18.81 vs. 110.25 ± 21.24 (?)	0.389 [−0.369; 1.148]	↑
			Pre‐ vs. post (CAD) (13)^§^	115.96 ± 12.01 vs. 118.34 ± 13.55 (?)	0.175 [−0.551; 0.900]	↔
			Pre‐ vs. post (C) (12)^§^	135.14 ± 15.43 vs. 133.54 ± 12.66 (?)	−0.105 [−0.855; 0.644]	↔
SV [mL]	Gayda et al. (2012)	?	Pre‐ vs. post (16)^#^	71 ± 21; 74 ± 17 (NS)	0.148 [−0.513; 0.809]	↔
			Pre‐ vs. 15‐min post (16)	71 ± 21 vs. 68 ± 20 (*P* < 0.05)	−0.139 [−0.800; 0.522]	↔
			Pre‐ vs. 120‐min post (16)	71 ± 21 vs. 73 ± 18 (NS)	0.097 [−0.564; 0.757]	↔
			C vs. S baseline (16)	69 ± 17 vs. 71 ± 21 (NS)	0.099 [−0.562; 0.759]	↔
			C vs. S 15‐min post (16)	70 ± 18 vs. 68 ± 20 (NS)	−0.100 [−0.760; 0.561]	↔
			C vs. S 120‐min post (16)	72 ± 19 vs. 73 ± 18 (NS)	0.051 [−0.609; 0.711]	↔
SVR [dyne s cm^−5^]	Gayda et al. (2012)	?	Pre‐ vs. post (16)^#^	1625 ± 417; 1145 ± 236 (*P* < 0.0001)	−1.262 [−1.974; −0.550]	↓↓↓^*^
			Pre‐ vs. 15‐min post (16)	823 ± 239 vs. 773 ± 255 (NS)	−0.192 [−0.854; 0.470]	↔
			Pre‐ vs. 120‐min post (16)	823 ± 239 vs. 873 ± 301 (NS)	0.173 [−0.489; 0.834]	↔
			C vs. S baseline (16)	813 ± 246 vs. 823 ± 239 (NS)	0.039 [−0.621; 0.699]	↔
			C vs. S 15‐min post (16)	896 ± 430 vs. 773 ± 255 (*P* < 0.0001)	−0.312 [−0.976; 0.350]	↓
			C vs. S 120‐min post (16)	961 ± 363 vs. 873 ± 301 (*P =* 0.0033)	−0.250 [−0.911; 0.413]	↓
24 h SBP [mmHg]	Gayda et al. (2012)	?	C vs. S (16)	136 ± 11 vs. 134 ± 9 (NS)	−0.188 [−0.849; 0.473]	↔
24 h DBP [mmHg]	Gayda et al. (2012)	?	C vs. S (16)	82 ± 6 vs. 81 ± 6 (NS)	−0.159 [−0.820; 0.503]	↔
24 h MBP [mmHg]	Gayda et al. (2012)	?	C vs. S (16)	100 ± 7 vs. 99 ± 7 (NS)	−0.136 [−0.797; 0.525]	↔
Metabolic blood biomarkers						
FFA [mmol L^−1^]	Pilch et al. (2010)^c^, ^$^	−	Pre‐ vs. post (10)	0.66 ± 0.30 vs. 0.72 ± 0.20 (NS)	0.210 [−0.602; 1.021]	↑
Glucose [mmol L^−1^]	Podstawski et al. (2021)	++	Pre‐ vs. post (30)	4.71 ± 0.93 vs. 4.15 ± 0.60 (*P* < 0.001)	−0.669 [−1.173; −0.164]	↓↓^*^
HDL‐C [mmol L^−1^]	Gryka et al. (2014)^$^, ^#^	−	Pre‐ vs. post (16)	1.29 ± 0.27 vs. 1.30 ± 0.27 (NS)	0.035 [−0.625; 0.695]	↔
	Pilch et al. (2010)^c^, ^$^	−	Pre‐ vs. post (10)	1.49 ± 0.30 vs.1.42 ± 0.20 (NS)	−0.245 [−1.056; 0.568]	↓
HDL‐C [mg dL^−1^]	Podstawski et al. (2021)	++	Pre‐ vs. post (30)	58.03 ± 14.82 vs. 65.37 ± 19.80 (*P =* 0.006)	0.401 [−0.099; 0.902]	↑
LDL‐C [mmol L^−1^]	Gryka et al. (2014)^$^	−	Pre‐ vs. post (16)	2.71 ± 0.47 vs. 2.67 ± 0.45 (NS)	−0.083 [−0.743; 0.577]	↔
	Pilch et al. (2010)^c^, ^$^	−	Pre‐ vs. post (10)	2.85 ± 0.80 vs. 2.78 ± 0.80 (NS)	−0.081 [−0.890; 0.728]	↔
LDL‐C [mg dL^−1^]	Podstawski et al. (2021)	++	Pre‐ vs. post (30)	100.23 ± 29.51 vs. 110.43 ± 29.22 (*P =* 0.007)	0.338 [−0.158; 0.835]	↑
TC [mmol L^−1^]	Gryka et al. (2014)^$^	−	Pre‐ vs. post (16)	4.50 ± 0.66 vs. 4.38 ± 0.62 (*P =* 0.007)	−0.178 [−0.840; 0.483]	↔
	Pilch et al. (2010)^c^, ^$^	−	Pre‐ vs. post (10)	4.64 ± 0.82 vs. 4.50 ± 0.80 (NS)	−0.160 [−0.970; 0.651]	↔
TC [mg dL^−1^]	Podstawski et al. (2021)	++	Pre‐ vs. post (30)	185.17 ± 35.93 vs. 198.27 ± 37.48 (*P =* 0.008)	0.348 [−0.150; 0.845]	↑
TG [mmol L^−1^]	Gryka et al. (2014)^$^	−	Pre‐ vs. post (16)	1.08 ± 0.43 vs, 0.91 ± 0.27 (*P =* 0.002)	−0.493 [−1.106; 0.228]	↓
	Pilch et al. (2010)^c^, ^$^	−	Pre‐ vs. post (10)	0.75 ± 0.20 vs. 0.66 ± 0.20 (NS)	−0.415 [−1.235; 0.404]	↓
TG [mg dL^−1^]	Podstawski et al. (2021)	++	Pre‐ vs. post (30)	123.97 ± 75.94; 112.4 ± 73.24 (NS)	−0.151 [−0.645; 0.343]	↔
Thermoregulation						
Forehead temperature [°C]	Podstawski et al. (2021)	++	Pre‐ vs. post (30)	36.53 ± 0.19 vs. 36.62 ± 0.56 (NS)	0.177 [−0.323; 0.678]	↔
*T*_re_ [°C]	Gryka et al. (2014)^$^	−	Pre‐ vs. post (16)	37.02 ± 0.2 vs. 38.56 ± 0.30 (*P =* 0.0004)	5.544 [3.630; 7.457]	↑↑↑^*^
	Gryka et al. (2020)^b^	+	Pre‐ vs. post (T) (10)	37.04 ± 0.18 vs. 38.51 ± 0.28 (*P* < 0.05)	5.521 [3.049; 7.994]	↑↑↑^*^
			Pre‐ vs. post (U) (10)	37.05 ± 0.23 vs. 38.66 ± 0.24 (*P* < 0.05)	6.319 [4.160; 8.479]	↑↑↑^*^
	Pilch et al. (2005)^$^	−	Pre‐ vs. post (T) (10)	36.7 ± ? vs. 38.1 ± ? (?)	N/A	N/A
			Pre‐ vs. post (U) (10)	36.9 ± ? vs. 38.3 ± ? (?)	N/A	N/A
	Pilch et al. (2007)^c^, ^$^	−	Pre‐ vs. post (10)	37.1 ± 0.3 vs. 38.1 ± 0.5 (*P* < 0.005)	2.118 [0.867; 3.368]	↑↑↑^*^
	Pilch et al. (2013)^$^	−	Pre‐ vs. post (A) (9)	37.01 ± 0.32 vs. 38.21 ± 0.32 (?)	3.429 [2.097; 4.760]	↑↑↑^*^
			Pre‐ vs. post (N) (9)	37.21 ± 0.37 vs. 38.41 ± 0.36 (?)	3.005 [1.786; 4.224]	↑↑↑^*^
	Pilch et al. (2014)^$^	−	Pre‐ vs. post (W) (10)	36.89 ± 0.12 vs. 38.50 ± 0.11 (*P* < 0.01)	12.886 [8.964; 16.809]	↑↑↑^*^
			Pre‐ vs. post (D) (10)	36.89 ± 0.13 vs. 38.05 ± 0.08 (*P* < 0.01)	9.427 [6.750; 12.105]	↑↑↑^*^
*T*_sub_ [°C]	Jezova et al. (1994)^#^	+	Pre‐ vs. post (F) (8)	36.39 ± 0.37 vs. 38.44 ± 0.37 (*P* < 0.01)	4.998 [3.059; 6.936]	↑↑↑^*^
			Pre‐ vs. 15‐min post (F) (8)	36.39 ± 0.37 vs. 36.31 ± 0.58 (NS)	−0.143 [−1.031; 0.745]	↔
			Pre‐ vs. 30‐min post (F) (8)	36.39 ± 0.37 vs. 36.10 ± 0.36 (NS)	−0.712 [−1.692; 0.207]	↓↓
			Pre‐ vs. post (M) (8)	36.26 ± 0.20 vs. 38.29 ± 0.54 (*P* < 0.01)	3.914 [0.585; 7.242]	↑↑↑^*^
			Pre‐ vs. 15‐min post (M) (8)	36.26 ± 0.20 vs. 36.22 ± 0.36 (NS)	−0.115 [−1.002; 0.773]	↔
			Pre‐ vs. 30‐min post (M) (8)	36.26 ± 0.20 vs. 36.22 ± 0.30 (NS)	−0.137 [−1.025; 0.750]	↔
Inflammatory response markers						
hsCRP [△%]	Kunutsor et al. (2018)^a^	+	Pre‐ vs. post (100)	1.71 (−0.38; 5.28) (*P =* 0.635)	N/A	N/A
			Pre‐ vs. 30‐min post (89)	0.62 (−1.40; 2.58) (*P =* 0.532)	N/A	N/A
HSP‐70 [ng mL^−1^]	Pilch et al. (2023)^b^, ^$^, ^#^	−	Pre‐ vs. post (T) (10)	0.025 ± 0.015 vs. 0.061 ± 0.040 (*P* < 0.05)	0.950 [−0.112; 2.010]	↑↑↑
			Pre‐ vs. post (U) (10)	0.021 ± 0.021 vs. 0.078 ± 0.050 (*P* < 0.05)	1.210 [0.089; 2.332]	↑↑↑^*^
IL‐1β [ng L^−1^]	Dugué & Léppanen (2000)	−	Pre‐ vs. post (20)	0.74 (ND; 8.5) vs. 0.68 (ND; 7.5) (NS)	N/A	N/A
IL‐1Ra [ng L^−1^]	Dugué & Léppanen (2000)	−	Pre‐ vs. post (20)	198 (100; 480) vs. 189 (105; 255) (NS)	N/A	N/A
IL‐6 [ng L^−1^]	Dugué & Léppanen (2000)	−	Pre‐ vs. post (20)	1.51 (0.55; 3.2) vs. 1.89 (0.72; 4.4) (*P* < 0.001)	N/A	N/A
IL‐6 [pg mL^−1^]	Pilch et al. (2023)^b^, ^$^, ^#^	−	Pre‐ vs. post (T) (10)	0.21 ± 0.09 vs. 0.37 ± 0.21 (*P* < 0.05)	0.810 [−0.147; 1.765]	↑↑↑
			Pre‐ vs. post (U) (10)	0.31 ± 0.26 vs. 0.68 ± 0.53 (*P* < 0.05)	0.744 [−0.162; 1.650]	↑↑
IL‐10 [pg mL^−1^]	Pilch et al. (2023)^b^, ^$^, ^#^	−	Pre‐ vs. post (T) (10)	0.94 ± 0.24 vs. 1.13 ± 0.25 (*P* < 0.05)	0.715 [−0.125; 1.555]	↑↑
			Pre‐ vs. post (U) (10)	0.99 ± 0.28 vs. 1.21 ± 0.44 (*P* < 0.05)	0.526 [−0.313; 1.366]	↑↑
Leukocyte count [10^3^ µL^−1^)	Dugué & Léppanen (2000)	−	Pre‐ vs. post (20)	6.6 (4.8; 9.2) vs. 7.7 (5.1; 11.0) (*P* < 0.001)	N/A	N/A
	Pilch et al. (2013)^$^	−	Pre‐ vs. post (A) (9)	4.60 ± 0.66 vs. 5.25 ± 0.82 (*P* < 0.05)	0.789 [−0.059; 1.638]	↑↑
			Pre‐ vs. post (N) (9)	5.24 ± 1.37 vs. 5.63 ± 1.10 (NS)	0.283 [−0.522; 1.089]	↑
	Pilch et al. (2023)^b^, ^$^, ^#^	−	Pre‐ vs. post (T) (10)	5.58 ± 1.41 vs. 6.24 ± 1.92 (*P* < 0.05)	0.354 [−0.466; 1.174]	↑
			Pre‐ vs. post (U) (10)	5.30 ± 0.95 vs. 5.85 ± 1.36 (NS)	0.420 [−0.406; 1.246]	↑
	Podstawski et al. (2021)	++	Pre‐ vs. post (30)	6.48 ± 1.24 vs. 6.29 ± 1.18 (NS)	−0.153 [−0.647; 0.341]	↔
Chronic						
Cardiovascular						
Antegrade shear rate [s^−1^]	Debray et al. (2023)^#^	++	Pre‐ vs. post (21)	60 ± 22 vs. 58 ± 30 (NS)	−0.071 [−0.655; 0.512]	↔
Baseline forearm blood flow [mL min^−1^]	Debray et al. (2023)	++	Pre‐ vs. post (21)	40 ± 20 vs. 34 ± 15 (NS)	−0.321 [−0.907; 0.265]	↓
Brachial artery diameter [mm]	Debray et al. (2023)	++	Pre‐ vs. post (21)	4.06 ± 0.88 vs. 3.92 ± 0.86 (*P =* 0.048)	−0.155 [−0.739; 0.429]	↔
Brachial artery FMD [%]	Debray et al. (2023)	++	Pre‐ vs. post (21)	4.02 ± 2.15 vs. 4.18 ± 2.82 (NS)	0.061 [−0.522; 0.644]	↔
cf‐PWV [m s^−1^]	Debray et al. (2023)^#^	++	Pre‐ vs. post (21)	8.67 ± 2.13 vs. 8.46 ± 1.58 (NS)	−0.106 [−0.689; 0.478]	↔
CVC_max_ at 44°C [U mmHg^−1^]	Debray et al. (2023)^#^	++	Pre‐ vs. post (21)	2.60 ± 1.18 vs. 2.58 ± 0.95 (NS)	−0.017 [−0.600; 0.565]	↔
CVC_nadir_ at 39°C [% of max CVC]	Debray et al. (2023)^#^	++	Pre‐ vs. post (21)	29.77 ± 15.61 vs. 28.90 ± 15.34 (NS)	−0.054 [−0.637; 0.529]	↔
CVC_peak_ at 39°C [% of max CVC]	Debray et al. (2023)^#^	++	Pre‐ vs. post (21)	45.57 ± 14.14 vs. 45.13 ± 17.51 (NS)	−0.026 [−0.609; 0.557]	↔
CVC_plateau_ at 39°C [% of max CVC]	Debray et al. (2023)^#^	++	Pre‐ vs. post (21)	59.29 ± 14.84 vs. 61.74 ± 21.67 (NS)	0.123 [−0.461; 0.707]	↔
DBP [mmHg]	Debray et al. (2023)^#^	++	Pre‐ vs. post (21)	72 ± 8 vs. 73 ± 8 (NS)	0.120 [−0.463; 0.704]	↔
			DiD (20)^§^	−4.08 ± 10.21 vs. −7.64 ± 9.33 (?)	0.699 [0.193; 1.205]	↑↑^*^
	Gryka et al. (2020)^b^	+	Pre‐ vs. post (T) (10)	80.0 ± 4.1 vs. 79.0 ± 5.7 (NS)	−0.181 [−0.993; 0.630]	↔
			DiD (T) (10)	−9.00 ± 6.72 vs. −6.00 ± 6.55 (NS)	−0.835 [−1.619; −0.051]	↓↓↓^*^
			Pre‐ vs. post (U) (10)	79.5 ± 4.4 vs. 80.5 ± 4.4 (NS)	0.210 [−0.602; 1.022]	↑
			DiD (U) (10)	−10.00 ± 6.01 vs. −8.50 ± 7.97 (NS)	−0.385 [−1.073; 0.303]	↓
	Pilch et al. (2007)^c^, ^$^	−	Pre‐ vs. post (10)^§^	72 ± 7.8 vs. 71 ± 8.3 (?)	−0.114 [−0.924; 0.695]	↔
			DiD (10)	−20.00 ± 16.18 vs. −16.00 ± 8.67 (NS)	−0.527 [−1.239; 0.185]	↓↓
FAVC_AUC_ [mL mmHg^−1^]	Debray et al. (2023)^#^	++	Pre‐ vs. post (21)	1.68 ± 1.20 vs. 1.54 ± 1.03 (*P =* 0.031)	−0.120 [−0.704; 0.478]	↔
FAVC_baseline_ [mL min^−1^ mmHg^−1^]	Debray et al. (2023)	++	Pre‐ vs. post (21)	0.47 ± 0.23 vs. 0.39 ± 0.16 (NS)	−0.378 [−0.965; 0.210]	↓
FAVC_peak_ [mL min^−1^ mmHg^−1^]	Debray et al. (2023)^#^	++	Pre‐ vs. post (21)	3.01 ± 1.48 vs. 2.72 ± 1.25 (*P =* 0.024)	−0.202 [−0.787; 0.382]	↓
HR [bpm]	Debray et al. (2023)^#^	++	Pre‐ vs. post (21)	57 ± 6 vs. 57 ± 7 (NS)	0.000 [−0.583; 0.583]	↔
			DiD (20)^§^	+17.80 ± 14.14 vs. +25.25 ± 13.47 (?)	−1037 [−1.603; −0.471]	↓↓↓^*^
	Gryka et al. (2014)^$^	−	Pre‐ vs. post (16)	69.00 ± 5.37; 68.25 ± 5.16 (NS)	−0.135 [−0.796; 0.525]	↔
			DiD (16)^§^	+63.00 ± 6.03 vs. +58.25 ± 5.00 (?)	1.619 [0.825; 2.412]	↑↑↑^*^
	Gryka et al. (2020)^b^	+	Pre‐ vs. post (T) (10)	67.6 ± 6.1 vs. 66.4 ± 3.4 (*P* < 0.05)	−0.210 [−1.020; 0.602]	↓
			DiD (T) (10)	+64.00 ± 5.82 vs. +58.00 ± 4.28 (*P* < 0.05)	2.120 [0.861; 3.380]	↑↑↑^*^
			Pre‐ vs. post (U) (10)	74.0 ± 6.9; 72.0 ± 5.7 (NS)	−0.289 [−1.102; 0.524]	↓
			DiD (U) (10)	+62.40 ± 8.65 vs. +57.60 ± 6.02 (NS)	1.154 [0.273; 2.035]	↑↑↑^*^
	Pilch et al. (2007)^c^, ^$^	−	Pre‐ vs. post (10)^§^	67 ± 10.55 vs. 61 ± 10.5 (?)	−0.526 [−1.351; 0.299]	↓↓
			DiD (10)	+51.00 ± 15.77 vs. +35.00 ± 10.11 (*P* < 0.005)	2.134 [0.869; 3.400]	↑↑↑^*^
Peak brachial artery diameter [mm]	Debray et al. (2023)	++	Pre‐ vs. post (21)	4.22 ± 0.87 vs. 4.08 ± 0.86 (*P =* 0.036)	−0.156 [−0.740; 0.428]	↔
Retrograde shear rate [s^−1^]	Debray et al. (2023)	++	Pre‐ vs. post (21)	−7 ± 7 vs. −8 ± 5 (NS)	−0.154 [−0.738; 0.430]	↔
SBP [mmHg]	Debray et al. (2023)^#^	++	Pre‐ vs. post (21)	115 ± 12 vs. 115 ± 12 (NS)	0.000 [−0.583; 0.583]	↔
			DiD (20)^§^	−8.31 ± 16.03 vs. −11.80 ± 15.23 (?)	0.429 [−0.043; 0.901]	↑
	Gryka et al. (2020)^b^	+	Pre‐ vs. post (T) (10)	124.0 ± 9.4 vs. 119.0 ± 9.7 (NS)	−0.483 [−1.306; 0.341]	↓
			DiD (T) (10)	+12.00 ± 9.11 vs. +13.00 ± 10.01 (NS)	−0.192 [−0.860; 0.475]	↔
			Pre‐ vs. post (U) (10)	116.5 ± 6.7 vs. 114.0 ± 8.1 (NS)	−0.307 [−1.124; 0.509]	↓
			DiD (U) (10)	+15.00 ± 7.56 vs. +12.00 ± 8.10 (NS)	0.706 [−0.045; 1.457]	↑↑
	Pilch et al. (2007)^c^, ^$^	−	Pre‐ vs. post (10)^§^	115 ± 9.8 vs. 112 ± 7.1 (?)	−0.316 [−1.130; 0.498]	↓
			DiD (10)	+13.00 ± 8.60 vs. +11.00 ± 8.91 (NS)	0.421 [−0.272; 1.115]	↑
Metabolic blood biomarkers						
FFA [mmol L^−1^]	Pilch et al. (2010)^c^, ^$^	−	Pre‐ vs. post (10)	0.66 ± 0.30 vs. 0.66 ± 0.40 (NS)	0.000 [−0.810; 0.810]	↔
			DiD (10)^§^	+0.06 ± 0.26 vs. +0.19 ± 0.40 (?)	−0.681 [−1.426; 0.064]	↓↓
Glycaemia [mmol L^−1^]	Debray et al. (2023)	++	Pre‐ vs. post (21)	5.59 ± 0.90 vs. 5.60 ± 1.1 (NS)	0.010 [−0.573; 0.593]	↔
HbA1c [%]	Debray et al. (2023)	++	Pre‐ vs. post (21)	5.9 ± 0.1 vs. 5.8 ± 0.6 (NS)	−0.173 [−0.791; 0.445]	↔
HDL‐C [mmol L^−1^]	Debray et al. (2023)	++	Pre‐ vs. post (21)	1.42 ± 0.45 vs. 1.48 ± 0.47 (NS)	0.125 [−0.458; 0.709]	↔
	Gryka et al. (2014)^$^, ^#^	−	Pre‐ vs. post (16)	1.29 ± 0.27 vs. 1.26 ± 0.26 (NS)	−0.108 [−0.769; 0.552]	↔
			DiD (16)^§^	+0.01 ± 0.27 vs. +0.10 ± 0.52 (?)	−0.381 [−0.908; 0.147]	↓
	Pilch et al. (2010)^c^, ^$^	−	Pre‐ vs. post (10)	1.49 ± 0.30 vs. 1.42 ± 0.3 (NS)	−0.215 [−1.027; 0.596]	↓
			DiD (10)^§^	−0.07 ± 0.26 vs. −0.03 ± 0.36 (?)	−0.228 [−0.899; 0.442]	↓
LDL‐C [mmol L^−1^]	Debray et al. (2023)	++	Pre‐ vs. post (21)	1.44 ± 0.81 vs. 1.39 ± 0.53 (NS)	−0.067 [−0.651; 0.516]	↔
	Gryka et al. (2014)^$^	−	Pre‐ vs. post (16)	2.71 ± 0.47 vs. 2.52 ± 0.43 (*P =* 0.013)	−0.401 [−1.068; 0.266]	↓
			DiD (16)^§^	−0.04 ± 0.46 vs. −0.08 ± 0.43 (?)	0.172 [−0.340; 0.683]	↔
	Pilch et al. (2010)^c^, ^$^	−	Pre‐ vs. post (10)	2.85 ± 0.80 vs. 2.50 ± 0.60 (NS)	−0.501 [−1.322; 0.319]	↓↓
			DiD (10)^§^	−0.07 ± 0.80 vs. −0.010 ± 0.60 (?)	0.077 [−0.585; 0.738]	↔
TC [mmol L^−1^]	Debray et al. (2023)	++	Pre‐ vs. post (21)	3.40 ± 1.03 vs. 3.42 ± 0.91 (NS)	0.020 [−0.563; 0.602]	↔
	Gryka et al. (2014)^$^	−	Pre‐ vs. post (16)	4.50 ± 0.66 vs. 4.25 ± 0.47 (*P =* 0.044)	−0.405 [−1.070; 0.261]	↓
			DiD (16)^§^	−0.12 ± 0.64 vs. −0.09 ± 0.51 (?)	−0.097 [−0.606; 0.411]	↔
	Pilch et al. (2010)^c^, ^$^	−	Pre‐ vs. post (10)	4.64 ± 0.82 vs. 4.25 ± 0.3 (*P* < 0.05)	−0.501 [−1.322; 0.319]	↓↓
			DiD (10)^§^	−0.14 ± 0.81 vs. −0.13 ± 0.61 (?)	−0.025 [−0.686; 0.635]	↔
TG [mmol L^−1^]	Debray et al. (2023)	++	Pre‐ vs. post (21)	1.18 ± 0.54 vs. 1.23 ± 0.61 (NS)	0.083 [−0.500; 0.667]	↔
	Gryka et al. (2014)^$^, ^#^	−	Pre‐ vs. post (16)	1.08 ± 0.43 vs. 1.06 ± 0.64 (NS)	−0.033 [−0.693; 0.627]	↔
			DiD (16)^§^	−0.17 ± 0.38 vs. −0.26 ± 0.56 (?)	0.346 [−0.178; 0.870]	↑
	Pilch et al. (2010)^c^, ^$^	−	Pre‐ vs. post (10)	0.75 ± 0.20 vs. 0.72 ± 0.20 (NS)	−0.138 [−0.949; 0.672]	↔
			DiD (10)^§^	−0.09 ± 0.20 vs. −0.01 ± 0.17 (?)	−0.785 [−1.556; −0.015]	↓↓^*^
Thermoregulation						
ΔPV [%]	Gryka et al. (2014)^$^	−	First vs. last exposure (16)	−8.13 ± 3.52 vs. −9.67 ± 2.93 (NS)	−0.450 [−1.117; 0.219]	↓
	Gryka et al. (2020)^b^	+	First vs. last exposure (T) (10)	−8.91 ± 3.85 vs. −11.17 ± 2.54 (*P* < 0.05)	−0.616 [−1.442; 0.211]	↓↓
			First vs. last exposure (U) (10)	−7.31 ± 2.52 vs. −7.91 ± 1.64 (NS)	−0.250 [−1.062; 0.562]	↓
	Pilch et al. (2007)^c^, ^$^	−	First vs. last exposure (10)	−4 ± 2.6 vs. −6.14 ± 5.3 (?)	−0.430 [−1.273; 0.413]	↓
Sweat rate [L h^−1^]	Debray et al. (2023)^#^	++	First vs. last exposure (19)	0.63 ± 0.42 vs. 0.93 ± 0.54 (*P =* 0.003)	0.587 [−0.043; 1.216]	↑↑
*T*_c_/*T*_rec_ [°C]	Debray et al. (2023)^#^	++	Pre‐ vs. post (19)	37.28 ± 0.45 vs. 37.01 ± 0.46 (*P =* 0.046)	−0.569 [−1.193; 0.055]	↓↓
			DiD (19)^§^	+0.44 ± 0.41 vs. +0.55 ± 0.40 (?)	−0.520 [−1.016; −0.025]	↓↓^*^
	Gryka et al. (2014)^$^	−	Pre‐ vs. post (16)	37.02 ± 0.2 vs. 36.65 ± 0.3 (*P* < 0.05)	−1.331 [−2.120; −0.543]	↓↓↓^*^
			DiD (16)^§^	+1.54 ± 0.26 vs. +1.48 ± 0.27 (?)	0.426 [−0.106; 0.958]	↑
	Gryka et al. (2020)^b^	+	Pre‐ vs. post (T) (10)	37.04 ± 0.18 vs. 36.68 ± 0.27 (NS)	−1.396 [−2.386; −0.404]	↓↓↓^*^
			DiD (T) (10)	+1.47 ± 0.25 vs. +1.41 ± 0.25 (NS)	0.448 [−0.250; 1.146]	↑
			Pre‐ vs. post (U) (10)	37.05 ± 0.23 vs. 36.64 ± 0.32 (NS)	−1.324 [−2.281; −0.367]	↓↓↓^*^
			DiD (U) (10)	+1.61 ± 0.24 vs. +1.56 ± 0.28 (NS)	0.351 [−0.332; 1.035]	↑
	Pilch et al. (2007)^c^, ^$^	−	Pre‐ vs. post (10)^§^	37.1 ± 0.3 vs. 37.0 ± 0.4 (?)	−0.256 [−1.071; 0.558]	↓
			DiD (10)	+1.00 ± 0.44 vs. +0.80 ± 0.36 (*P* < 0.05)	0.915 [0.109; 1.721]	↑↑↑^*^
*T*_sk_ [°C]	Debray et al. (2023)^#^	++	Pre‐ vs. post (20)^§^	31.63 ± 2.57 vs. 31.82 ± 0.91 (?)	0.081 [−0.516; 0.677]	↔
			DiD (20)^§^	+8.01 ± 2.23 vs. +8.55 ± 1.00 (?)	−0.528 [−1.011; −0.045]	↓↓^*^
Inflammatory response markers						
HSP‐70 [ng mL^−1^]	Pilch et al. (2023)^b^, ^$^, ^#^	−	Pre‐ vs. post (T) (10)	0.025 ± 0.015 vs. 0.029 ± 0.020 (NS)	0.205 [−0.608; 1.017]	↑
			DiD (T) (10)^§^	+0.036 ± 0.040 vs. +0.012 ± 0.03 (?)	1.402 [0.433; 2.371]	↑↑↑^*^
			Pre‐ vs. post (U) (10)	0.021 ± 0.021 vs. 0.033 ± 0.010 (NS)	0.609 [−0.217; 1.434]	↑↑
			DiD (U) (10)^§^	+0.057 ± 0.040 vs. +0.009 ± 0.030 (?)	2.341 [0.985; 3.697]	↑↑↑^*^
IL‐6 [pg mL^−1^]	Pilch et al. (2023)^b^, ^$^, ^#^	−	Pre‐ vs. post (T) (10)	0.21 ± 0.09 vs. 0.29 ± 0.27 (NS)	0.310 [−0.538; 1.158]	↑
			DiD (T) (10)^§^	+0.16 ± 0.18 vs. +0.09 ± 0.30 (?)	0.487 [−0.218; 1.192]	↑
			Pre‐ vs. post (U) (10)	0.31 ± 0.26 vs. 0.50 ± 0.39 (NS)	0.510 [−0.326; 1.346]	↑↑
			DiD (U) (10)^§^	+0.37 ± 0.46 vs. +0.11 ± 0.40 (?)	1.115 [0.246; 1.983]	↑↑↑^*^
IL‐10 [pg mL^−1^]	Pilch et al. (2023)^b^, ^$^, ^#^	−	Pre‐ vs. post (T) (10)	0.94 ± 0.24 vs. 1.13 ± 0.30 (NS)	0.683 [−0.202; 1.478]	↑↑
			DiD (T) (10)^§^	+0.19 ± 0.25 vs. +0.22 ± 0.29(?)	−0.205 [−0.873; 0.464]	↓
			Pre‐ vs. post (U) (10)	0.99 ± 0.28 vs. 1.08 ± 0.29 (NS)	0.292 [−0.523; 1.106]	↑
			DiD (U) (10)^§^	+0.22 ± 0.39 vs. +0.26 ± 0.32 (?)	−0.207 [−0.876; 0.462]	↓
Leukocyte count [10^3^ µL^−1^]	Pilch et al. (2023)^b^, ^$^, ^#^	−	Pre‐ vs. post (T) (10)	5.58 ± 1.41 vs. 5.53 ± 1.62 (NS)	−0.030 [−0.839; 0.779]	↔
			DiD (T) (10)^§^	+0.66 ± 1.72 vs. +0.34 ± 1.73 (?)	0.342 [−0.340; 1.025]	↑
			Pre‐ vs. post (U) (10)	5.30 ± 0.95 vs. 5.20 ± 0.85 (NS)	−0.102 [−0.912; 0.708]	↔
			DiD (U) (10)^§^	+0.55 ± 1.21 vs. +0.33 ± 0.83 (?)	0.379 [−0.308; 1.067]	↑

Abbreviations: AIx, augmentation index; AP, arterial pressure; (cf)PWV, (carotid‐femoral) pulse wave velocity; CO, cardiac output; CVC, cutaneous vascular conductance; DT, diastolic time; EDV, end‐diastolic volume; FAVC, forearm vascular conductance during reactive hyperaemia; FFA, free fatty acid; FMD, flow‐mediated dilation; HbA1c, glycated haemoglobin; Hct, haematocrit; HDL‐C, high‐density lipoprotein cholesterol; hsCRP, high sensitivity C‐reactive protein; HSP‐70, heat shock protein 70; IL, interleukin; LDL‐C, low‐density lipoprotein cholesterol; (L)VET, (left‐)ventricular ejection time; MAP, mean arterial pressure; PP(a), pulse pressure (amplification); PV, plasma volume; SV, stroke volume; SVR, systemic vascular resistance; TC, total cholesterol; T_c_, core temperature; TG, triglyceride; T_re_, rectal temperature; T_sk_, skin temperature; T_sub_, sublingual temperature.

^a,b,c^ Papers derived from the same study.

^$^ Sample size stated in the methods was used, as it was not reported for this outcome.

^#^ (Some) data were extracted from graphs using PlotDigitizer.

^§^ *P*‐value not reported; analysis not from original study.

* Significant effect based on the confidence interval.

++: low risk of bias; +: low risk of bias with concerns about uncontrolled confounding; ?: unclear risk of bias; −: high risk of bias.

↑/↓, ↑↑/↓↓, ↑↑↑/↓↓↓ = low, medium, large effect size; ↔ = no effect.

#### 3.4.1 Cardiovascular

##### BP

**FIGURE 2:**
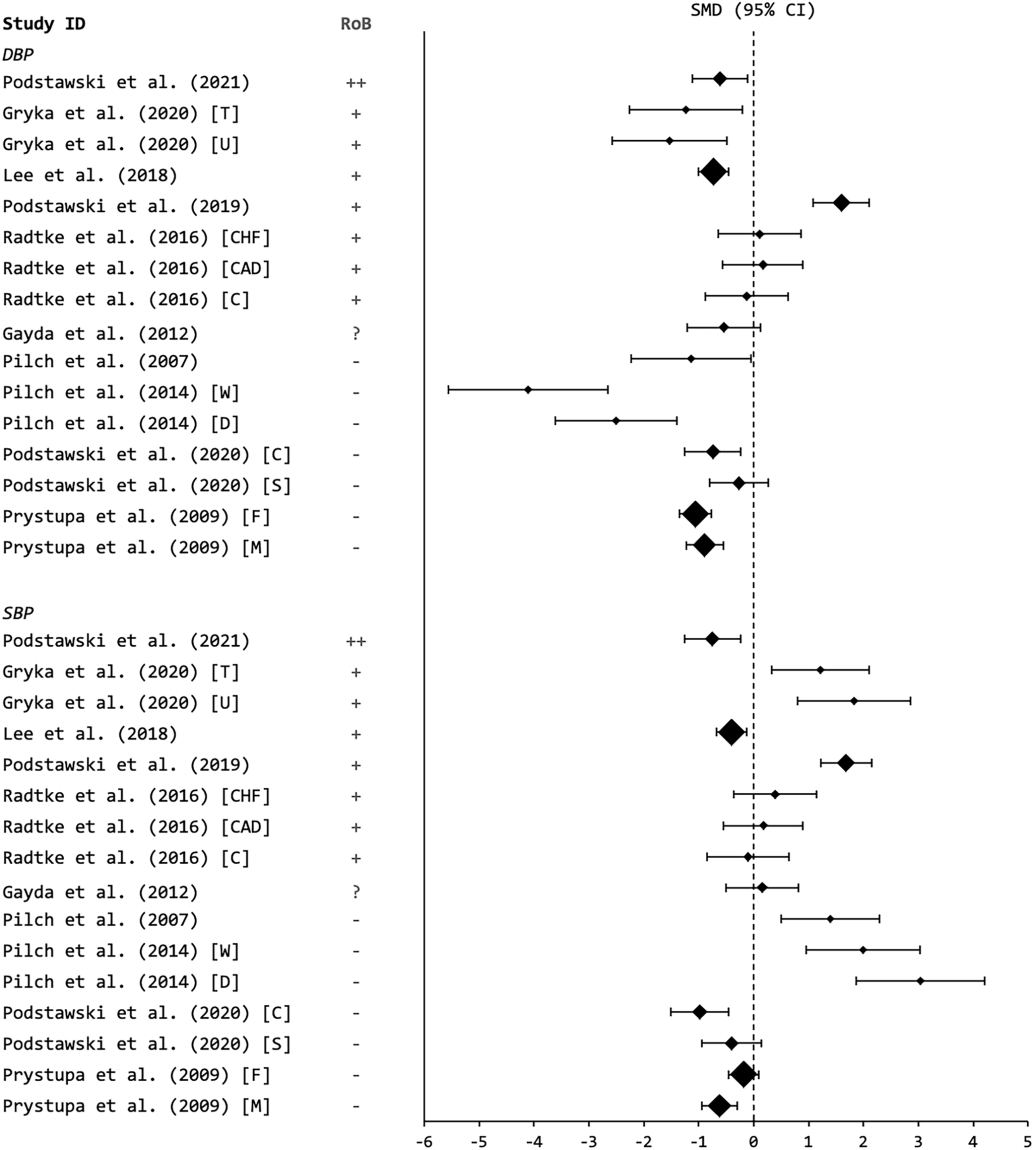
Acute effects of contrast therapy on BP. RoB indicates the risk‐of‐bias assessment. Diamonds represent SMDs, with horizontal lines indicating 95% CIs. Positive values indicate an increase relative to baseline, negative values indicate a decrease.

Acute contrast exposure generally led to decreases in diastolic blood pressure (DBP) (Gryka et al., 2020; Lee et al., 2018; Pilch et al., 2007, 2014; Podstawski et al., 2020, 2021; Prystupa et al., 2009), with reductions ranging from −7 to −20 mmHg (SMD: −0.735 to −4.111; Figure 2). Conversely, one study reported an increase in DBP (Podstawski et al., 2019). Other studies reported varying changes, ranging from −4.00 to +1.59 mmHg (SMD: −0.544 to 0.163; Figure 2) (Gayda et al., 2012; Podstawski et al., 2020; Radtke et al., 2016). No subacute changes or differences relative to a control group were observed (Gayda et al., 2012; Lee et al., 2018).

Acute contrast exposure led to increases (Gryka et al., 2014, 2020; Pilch et al., 2007; Podstawski et al., 2019) and decreases (Podstawski et al., 2020, 2021; Prystupa et al., 2009) in systolic blood pressure (SBP), ranging from +12 to +20 mmHg (SMD: 1.216 to 3.034; Figure 2) and −8.5 to −9.7 mmHg (SMD: −0.624 to −0.985; Figure 2), respectively. Other studies reported mixed changes, ranging from −6.20 to +8.38 mmHg (SMD: −0.406 to 0.389; Figure 2) (Gayda et al., 2012; Lee et al., 2018; Podstawski et al., 2020; Radtke et al., 2016). One study reported lowered values 30 min post‐exposure (Lee et al., 2018). The assessment of 24‐h systolic, diastolic and mean BP showed no changes (Gayda et al., 2012).

##### HR

**FIGURE 3:**
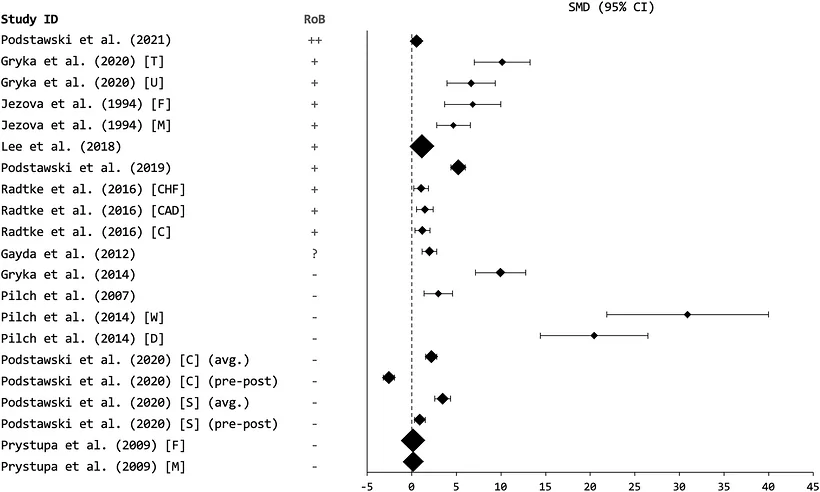
Acute effects of contrast therapy on HR. RoB indicates the risk‐of‐bias assessment. Diamonds represent SMDs, with horizontal lines indicating 95% CIs. Positive values indicate an increase relative to baseline, negative values indicate a decrease.

Acute contrast exposure predominantly increased HR (Gayda et al., 2012; Gryka et al., 2014, 2020; Jezova et al., 1994; Lee et al., 2018; Pilch et al., 2007, 2014; Podstawski et al., 2019, 2020, 2021; Radtke et al., 2016), ranging from +7.43 to +72.00 bpm (SMD: 0.545 to 30.917; Figure 3). In contrast, one study also reported a decrease (Podstawski et al., 2020). Subacutely, HR tended to be higher in men in one study (Table 3) (Jezova et al., 1994). Other subacute differences ranged from −4.00 to +2.01 bpm (SMD: −0.362 to 0.283; Table 3) (Gayda et al., 2012; Jezova et al., 1994; Lee et al., 2018). Relative to a control condition, HR also tended to be higher at 15 min post‐exposure (Gayda et al., 2012).

##### Vascular health

Acute exposure was associated with decreases in augmentation index (AIx), diastolic time, left‐ventricular ejection time, mean arterial pressure (MAP), and vascular resistance, with left‐ventricular ejection time and MAP tending to remain lowered subacutely (Table 3) (Gayda et al., 2012; Lee et al., 2018). Pulse wave velocity (PWV) decreased acutely and remained lower at 30 min post‐exposure, whereas pulse pressure tended to increase acutely and was reduced subacutely (Table 3) (Lee et al., 2018). Cardiac output increased acutely, with no subacute effects or differences relative to a control group (Table 3) (Gayda et al., 2012; Radtke et al., 2016). No changes were reported for arterial pressure, pulse pressure amplification (Lee et al., 2018), stroke volume or end‐diastolic volume (Table 3) (Gayda et al., 2012).

#### 3.4.2 Metabolic blood biomarkers

##### Glucose metabolism

Glucose concentrations were reported to decrease following acute contrast exposure (Table 3) (Podstawski et al., 2021).

##### Fat metabolism

**FIGURE 4:**
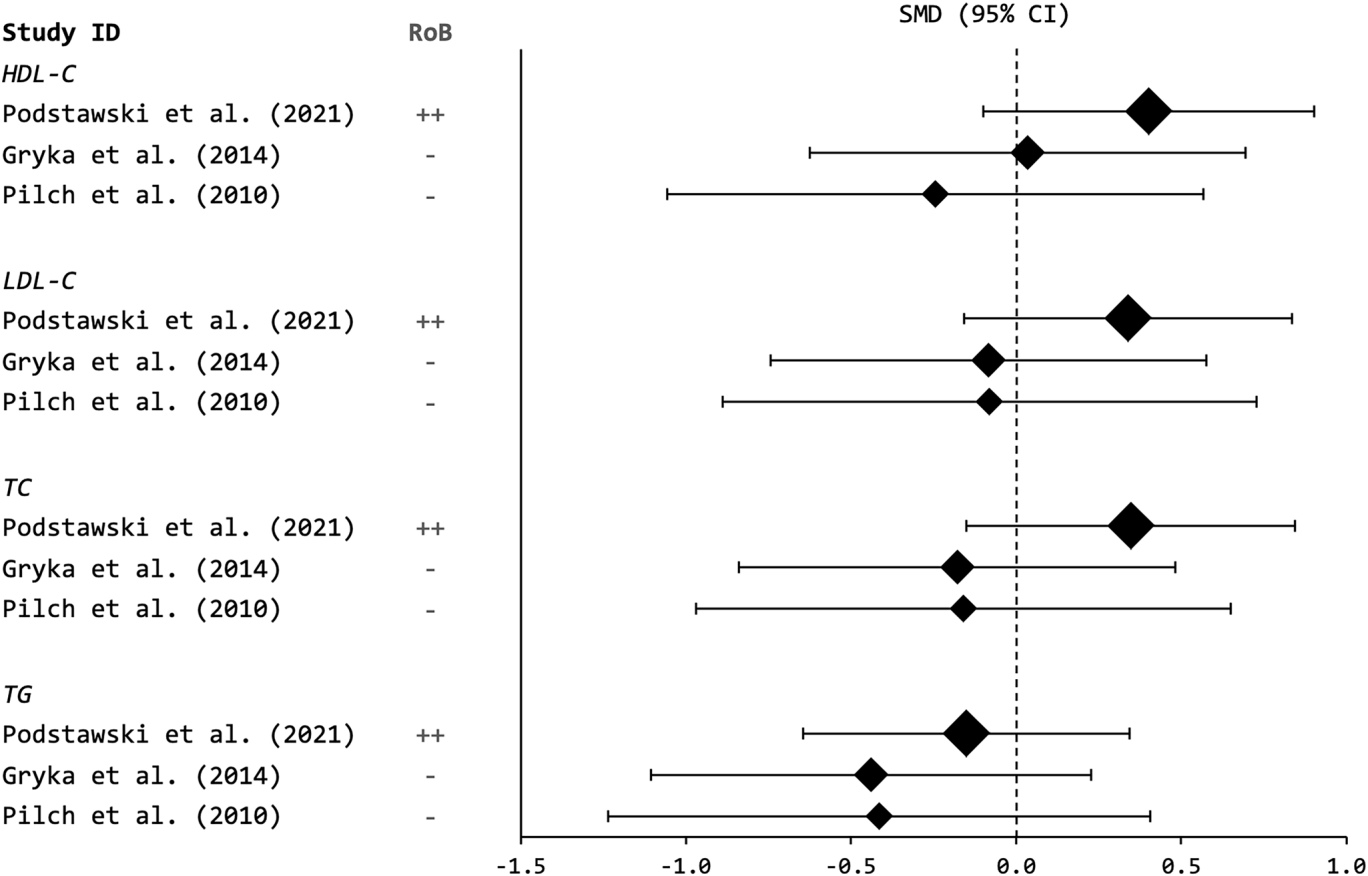
Acute effects of contrast therapy on fat metabolism. RoB indicates the risk‐of‐bias assessment. Diamonds represent SMDs, with horizontal lines indicating 95% CIs. Positive values indicate an increase relative to baseline, negative values indicate a decrease.

High‐density lipoprotein–cholesterol (HDL‐C), low‐density lipoprotein–cholesterol (LDL‐C) and total cholesterol (TC) concentrations tended to increase in one study (Figure 4) (Podstawski et al., 2021). In contrast, no significant changes were observed in free fatty acids (FFA), HDL‐C, LDL‐C, TC, or triglycerides (TGs) in other studies (Figure 4; Table 3) (Gryka et al., 2014; Pilch et al., 2010; Podstawski et al., 2021).

#### 3.4.3 Thermoregulation

**FIGURE 5:**
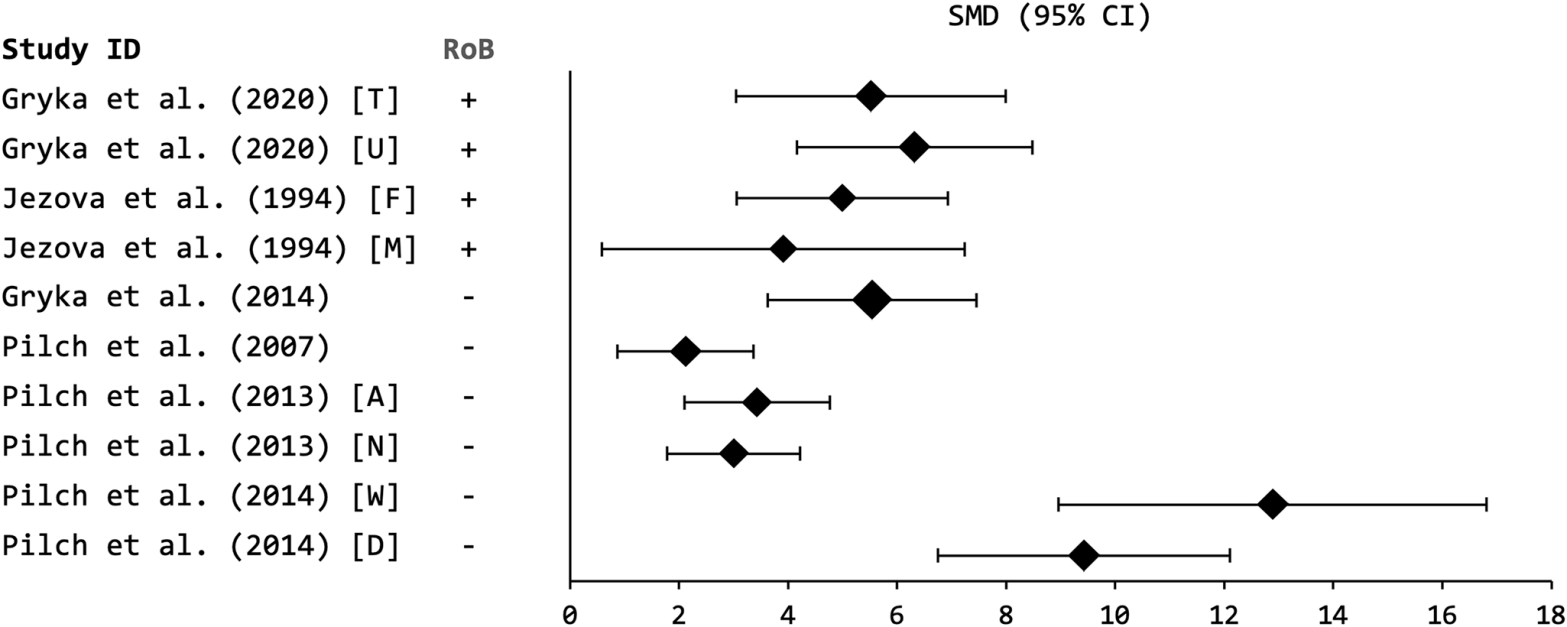
Acute effects of contrast therapy on measures of core temperature. RoB indicates the risk‐of‐bias assessment. Diamonds represent SMDs, with horizontal lines indicating 95% CIs. Positive values indicate an increase relative to baseline values.

Core temperature, assessed via rectal, sublingual and tympanic measures, increased upon acute contrast exposure (Gryka et al., 2014, 2020; Jezova et al., 1994; Pilch et al., 2007 2013, 2014), while forehead temperature was unchanged (Podstawski et al., 2021). Acute increases ranged from +1.00 to +2.05°C (SMD: 2.118 to 12.886; Figure 5). Subacutely, sublingual temperature tended to be lowered in women (Jezova et al., 1994).

#### 3.4.4 Inflammatory response markers

Heat shock protein (HSP)‐70, interleukin (IL)‐6 and IL‐10 concentrations increased or tended to increase (Pilch et al., 2023). Similarly, leukocyte count tended to increase in one study in athletes (Pilch et al., 2013), but remained unchanged in others (Pilch et al., 2013, 2023; Podstawski et al., 2021). These changes ranged from −0.19 to +0.66 × 10^3^ µL^−1^ (SMD: −0.153 to 0.789; Table 3).

### 3.5 Chronic effects of contrast therapy

The chronic effects of contrast therapy are summarised in Table 3. None of the included studies reported SMDs, which were therefore calculated (see Methods, Data synthesis, Appendix B).

#### 3.5.1 Cardiovascular

##### BP

**FIGURE 6:**
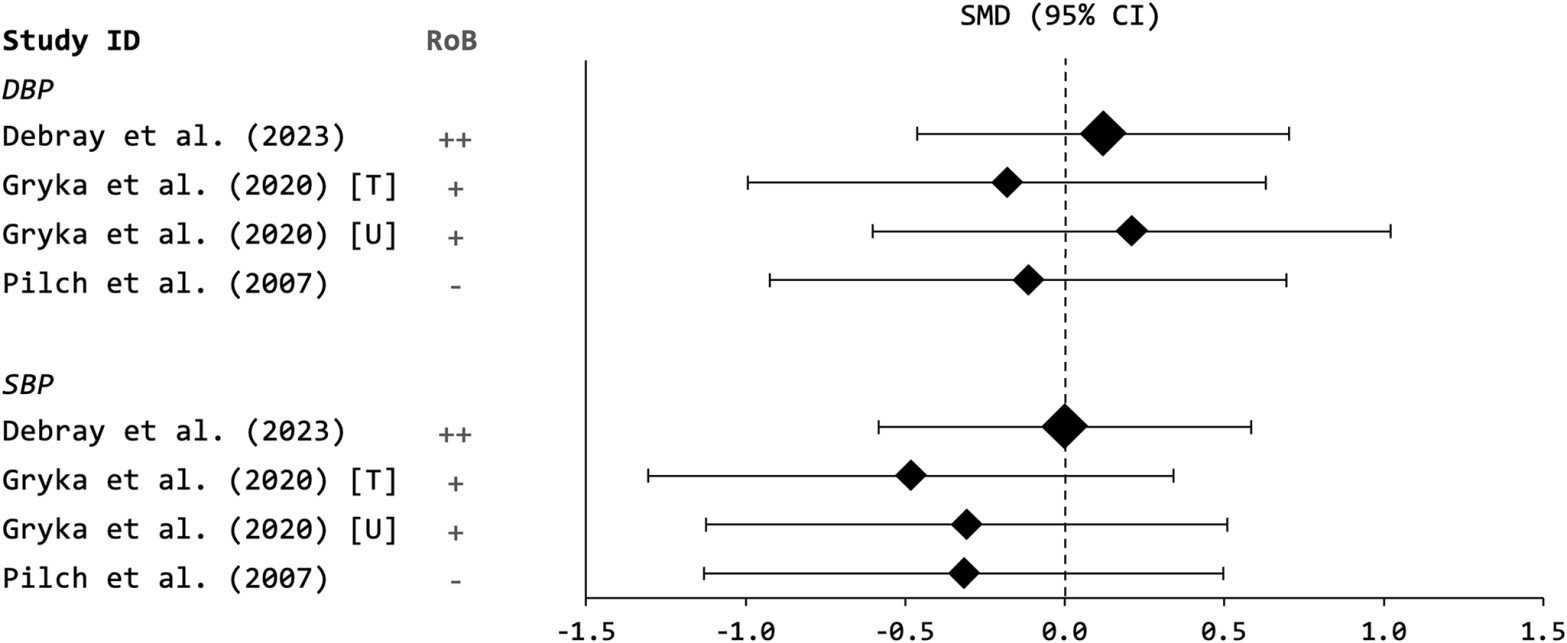
Chronic effects of contrast therapy on resting BP. RoB indicates the risk‐of‐bias assessment. Diamonds represent SMDs, with horizontal lines indicating 95% CIs. Positive values indicate an increase relative to pre‐intervention, negative values indicate a decrease.

No significant effects of repeated contrast therapy on resting DBP (−1 to 1 mmHg; Table 3) or SBP (−5 to 0 mmHg; Table 3) were reported (Figure 6) (Debray et al., 2023; Gryka et al., 2020; Pilch et al., 2007). Acute changes during repeated exposures were variable. One study reported a greater decrease in DBP during the final exposure compared with the first (Debray et al., 2023). Others reported smaller or unchanged values, with change score differences ranging from 1.5 to 4.0 mmHg (SMD: −0.385 to −0.835; Table 3) (Gryka et al., 2020; Pilch et al., 2007). Similarly, tendencies were reported for a greater acute reduction in SBP during the final exposure (Debray et al., 2023) and a smaller acute increase (Gryka et al., 2020).

##### HR

**FIGURE 7:**
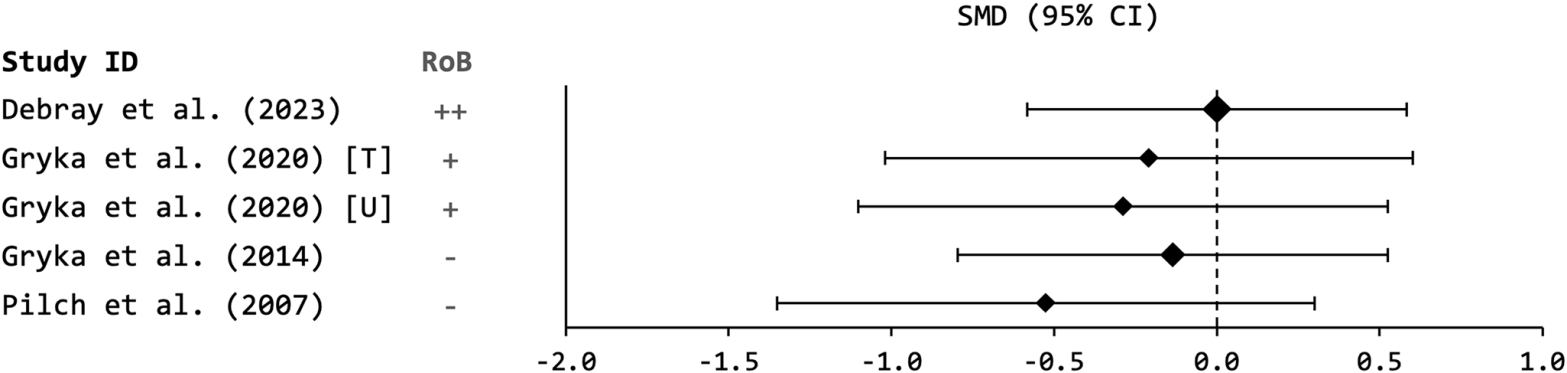
Chronic effects of contrast therapy on resting HR. RoB indicates the risk‐of‐bias assessment. Diamonds represent SMDs, with horizontal lines indicating 95% CIs. Positive values indicate an increase relative to pre‐intervention, negative values indicate a decrease.

Resting HR showed a tendency for reduction in one study (Pilch et al., 2007), but was generally unaffected by chronic contrast exposure (−6 to 0 bpm; Table 3) (Figure 7) (Debray et al., 2023; Gryka et al., 2014, 2020). The acute response predominantly showed smaller increases in HR during the final exposure compared with the first, with differences ranging from 4.75 to 16.00 bpm (SMD: 1.154 to 2.134; Table 3) (Gryka et al., 2014, 2020; Pilch et al., 2007). In contrast, one study reported a greater increase during the final exposure (Table 3) (Debray et al., 2023).

##### Vascular health

Evidence on the effects of chronic contrast therapy on vascular health is limited to a single study (Debray et al., 2023). No significant changes were found for measures of endothelial function, arterial stiffness or skin microvascular function (Table 3).

#### 3.5.2 Metabolic blood biomarkers

##### Glucose metabolism

Evidence on the effects of repeated contrast therapy on glucose metabolism is restricted to a single study (Debray et al., 2023), which compared resting glycaemia and HbA1c values before and after the intervention period. Both outcomes were unchanged (Table 3).

##### Fat metabolism

**FIGURE 8:**
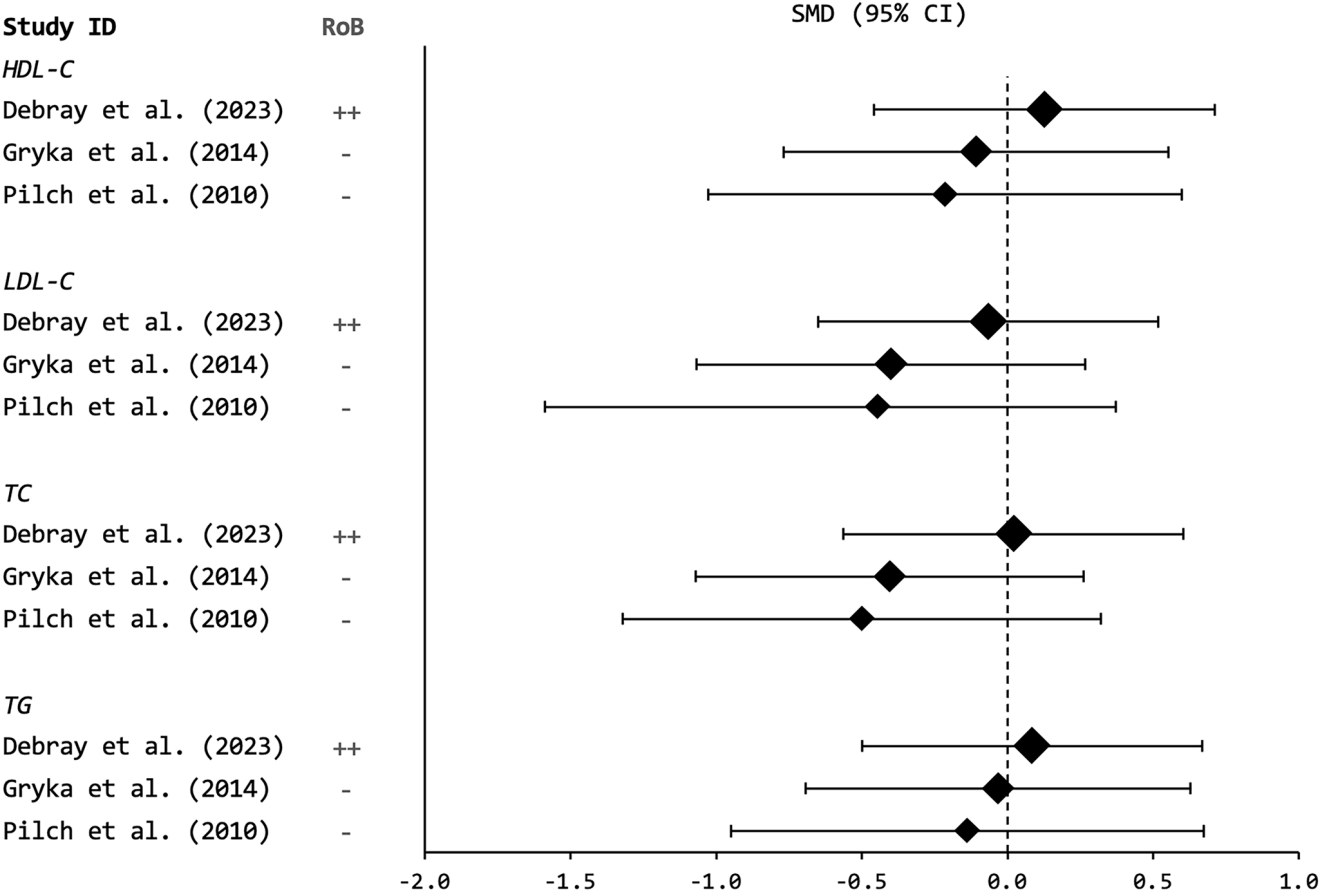
Chronic effects of contrast therapy on resting fat metabolism. RoB indicates the risk‐of‐bias assessment. Diamonds represent SMDs, with horizontal lines indicating 95% CIs. Positive values indicate an increase relative to pre‐intervention, negative values indicate a decrease.

Baseline concentrations of FFAs, HDL‐C, LDL‐C, TC and TGs were unchanged (Figure 8; Table 3) (Debray et al., 2023; Gryka et al., 2014; Pilch et al., 2010). Compared with the first exposure, the final exposure showed a tendency for a smaller decrease in TGs and a greater increase in FFAs (Table 3) (Pilch et al., 2010). No other changes in the acute responses of HDL‐C, LDL‐C, TC or TG were reported (Debray et al., 2023; Gryka et al., 2014; Pilch et al., 2010).

#### 3.5.3 Thermoregulation

**FIGURE 9:**
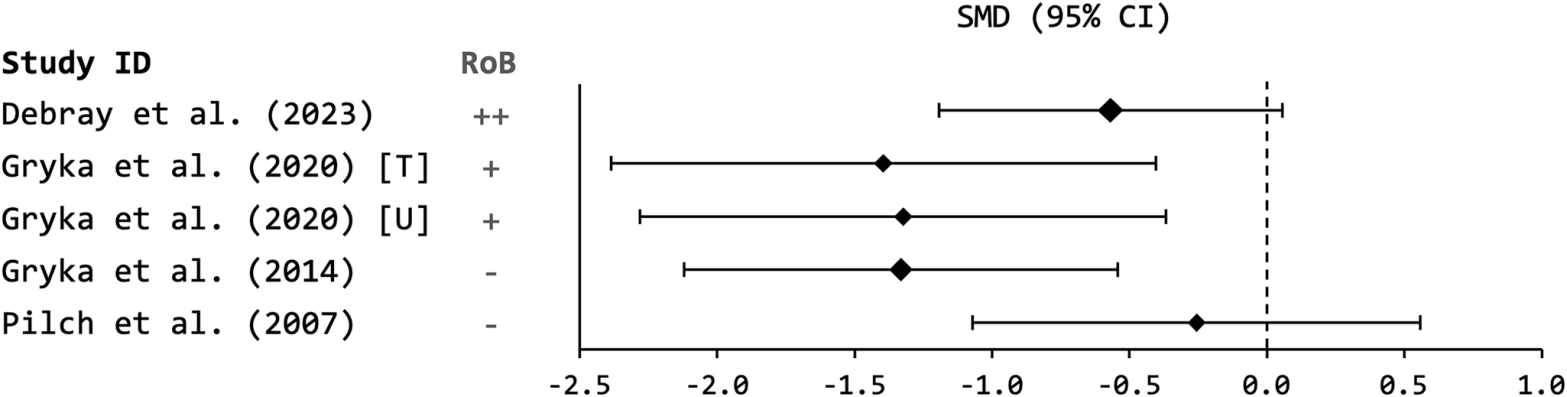
Chronic effects of contrast therapy on resting core temperature. RoB indicates the risk‐of‐bias assessment. Diamonds represent SMDs, with horizontal lines indicating 95% CIs. Positive values indicate an increase relative to pre‐intervention, negative values indicate a decrease.

Resting core and rectal temperatures generally decreased or tended to decrease following repeated contrast therapy (Debray et al., 2023; Gryka et al., 2014, 2020), ranging from −0.41 to −0.10°C (SMD: −1.396 to −0.256; Figure 9). Resting skin temperature remained unchanged (Debray et al., 2023). Acute responses during repeated exposures were more variable. Compared with the first exposure, increases in core and rectal temperature were smaller in most studies, ranging from 0.06 to 0.20°C (SMD: 0.426 to 0.915; Table 3) (Gryka et al., 2014; Pilch et al., 2007). Other studies reported no change in the acute response (Gryka et al., 2020), or greater increases in both core and skin temperature (Debray et al., 2023).

Other thermoregulatory measures were also inconsistent. Sweat rate tended to be higher during the final exposure (Debray et al., 2023). Decreases in plasma volume (PV) were generally unchanged (Gryka et al., 2014; 2020; Pilch et al., 2007), with differences compared to the first exposure ranging from −0.60% to −2.26% (SMD: −0.250 to −0.616; Table 3).

#### 3.5.4 Inflammatory response markers

Reports on inflammatory markers following chronic contrast therapy are limited to a single study (Pilch et al., 2023). Baseline concentrations of HSP‐70, IL‐6 and IL‐10 tended to increase or remain unchanged, whereas leukocyte count was consistently unchanged. Acute increases in HSP‐70 and IL‐6 were smaller during the final exposure compared with the first, while no changes in the acute responses of IL‐10 and leukocyte count were observed.

## 4 DISCUSSION

This review aimed to investigate the effects of acute and chronic contrast therapy on cardiometabolic health markers. Overall, the available evidence suggests that repeated contrast therapy may induce thermoregulatory and cardiovascular adaptations, as reflected by reductions in resting core temperature and attenuated acute cardiovascular and thermal responses. Evidence regarding metabolic outcomes was limited. Only a single study assessed acute glucose responses, a single study assessed chronic glycaemic outcomes, and three studies reported lipid responses, thereby limiting the conclusions that can be drawn regarding the metabolic effects of contrast therapy. Finally, acute contrast therapy appeared to elicit transient increases in inflammatory markers, whereas no chronic inflammatory adaptations were identified.

### 4.1 Thermoregulatory and cardiovascular effects

Collectively, the thermoregulatory and cardiovascular findings suggest that repeated contrast exposure may induce physiological adaptations consistent with heat acclimation (HA). However, the evidence varies across outcomes, and these adaptations seem to be only partially developed.

The reported decreases in resting core temperature, together with attenuated acute core temperature responses following chronic contrast exposure (Table 3; Figure 9), are consistent with findings reported in HA studies (Brunt et al., 2016; Ely et al., 2019; Leppaluoto et al., 1986; Pallubinsky et al., 2017, 2020; Ravanelli et al., 2021). Similar reductions in resting core temperature (−0.1 to −0.5°C) have been shown to correlate with smaller increases in core temperature during subsequent heat exposure (Buono et al., 1998; Kampmann et al., 2008). This suggests that a lower resting core temperature might contribute to reduced physiological strain during heat exposure and improved heat tolerance (Buono et al., 1998; Taylor, 2014). While reductions in resting core temperature were consistently reported across studies, evidence for attenuated acute core temperature responses was less consistent; one low‐risk‐of‐bias study reported no effect. Nevertheless, the overall pattern of findings may indicate that the adaptive response was predominantly driven by the heat stimulus, despite the inclusion of repeated cooling phases.

Following chronic contrast exposure, sweat rate also tended to be higher during the final exposure (Debray et al., 2023), representing another feature of HA and enhanced thermoregulatory efficiency. In contrast, acute reductions in PV remained unchanged following chronic contrast exposure (Gryka et al., 2014, 2020; Pilch et al., 2007), suggesting that not all thermophysiological adaptations typically associated with HA (Brunt & Minson, 2021; Taylor, 2014) were evident following repeated contrast exposure.

Acute contrast exposure places a substantial cardiovascular demand on the body, indicated by increased HR and cardiac output, shortened left ventricular ejection and diastolic time, and changes in BP, vascular resistance, and arterial stiffness (Table 3; Figures 2 and 3). With repeated contrast exposure, acute BP and HR responses were attenuated, with smaller decreases in DBP and smaller increases in SBP and HR (Table 3). Similar to thermoregulatory adaptations observed in core temperature responses, these findings suggest a reduced physiological strain following repeated contrast exposure.

The direction and magnitude of acute BP responses to thermal exposure likely depend on the relative dominance of heat‐ versus cold‐induced effects. Reported acute post‐contrast‐exposure reductions in DBP and SBP were often observed in protocols in which heating phases were longer, more intense or concluded the session (Lee et al., 2018; Pilch et al., 2014; Podstawski et al., 2020; Prystupa et al., 2009). Although protocols ending with rapid or intense cooling might be expected to evoke transient increases in BP, this was not consistently observed (Podstawski et al., 2020; Prystupa et al., 2009). One possible explanation is that such increases may be short‐lived and therefore not always captured depending on measurement timing. For instance, assessments conducted a few moments after cold exposure may miss an immediate but transient pressor response (Podstawski et al., 2020; Prystupa et al., 2009). Moreover, the cold stimulus used in contrast protocols is often milder than that employed in classic cold‐exposure studies (Sellers et al., 2021), and may therefore elicit smaller or even negligible pressor responses. In addition, these responses may also be influenced by participant characteristics, including training status and habitual sauna exposure (Podstawski et al., 2019), as well as environmental factors such as humidity, which can impair evaporative heat loss and amplify cardiovascular strain (Pilch et al., 2014).

Variability in acute HR responses across studies likely reflects differences in thermal load, participant characteristics and recovery periods between heating bouts (Podstawski et al., 2019, 2020). Short recovery phases (2–5 min), including brief cooling or showering, may be insufficient to restore baseline HR, whereas more prolonged CWI (2 min at 11–12°C) appears more effective (Podstawski et al., 2020).

Despite evidence of partial adaptation similar to HA, resting BP and HR remained unchanged following chronic contrast exposure (Table 3; Figures 6 and 7), and evidence of longer‐term improvements in vascular health was limited (Debray et al., 2023). A potential explanation is that intermittent cooling phases within contrast protocols may reduce the cumulative heat strain compared with traditional HA protocols. This may result in smaller elevations in core temperature and a reduced overall thermal stimulus. Conversely, HA protocols generally employ either sustained heat exposure or controlled hyperthermia (e.g., clamping core temperature) to ensure a more prolonged and consistent heat load sufficient to induce physiological adaptations (Brunt et al., 2016; Ely et al., 2019; James et al., 2023; Taylor, 2014). Consequently, the thermal stimulus in contrast therapy protocols might have been insufficient to elicit the full spectrum of HA responses.

### 4.2 Metabolic adaptations

Evidence regarding the impact of contrast exposure on metabolic health remains limited and is insufficient to draw firm conclusions. Nonetheless, the available findings may provide some preliminary insight into the potential metabolic effects of contrast exposure. Following acute contrast exposure, one study reported reduced glucose concentrations and trends towards small‐to‐medium increases in HDL‐C, LDL‐C and TC (Podstawski et al., 2021). No changes in baseline lipid concentrations, resting glycaemia or HbA1c were reported after repeated contrast exposure (Table 3; Figure 8). Notably, the only study reporting acute metabolic changes was judged to be at low risk of bias and of higher methodological quality than the studies reporting null lipid findings (Podstawski et al., 2021).

The observed acute reduction in glucose concentrations is more consistent with favourable metabolic responses reported following acute cold exposure than with heat exposure. Heat exposure is generally associated with impaired glucose regulation, evidenced by elevated FPG and/or reduced glucose tolerance during and following exposure (Behzadi et al., 2022; Kimball et al., 2018). Conversely, acute cold exposure effects are varying, with favourable effects, enhancing insulin sensitivity and reducing fasting and postprandial insulin levels, and reducing FPG in individuals with obesity or T2D (Blondin et al., 2015, 2017, 2020), while increased glucose and insulin concentrations have also been noted alongside decreased insulin sensitivity (Sellers et al., 2021). One proposed mechanism for cold‐induced improvements in glycaemia is GLUT4 translocation (Ivanova & Blondin, 2021). Although GLUT4 contents were not assessed, the 1‐min CWI (10–11°C) cooling breaks may have increased translocation, which could partly explain the reductions in glucose levels reported by Podstawski et al. (2021).

The absence of improvements in glycaemia following chronic contrast exposure differs from several studies of isolated heat and cold interventions. Chronic heat exposure has been associated with reduced FPG levels and improved glucose handling (Ely et al., 2019; Imamura et al., 2001; Pallubinsky et al., 2020), although null effects on FPG and postprandial glucose handling have also been reported (James et al., 2023). It has been suggested that studies reporting no meaningful effects may have implemented insufficient heat stress, characterised by exposures that were too short or insufficiently intense (James et al., 2023). This is consistent with the hypothesis that heat‐induced improvements in glucose regulation may be mediated by the HSP response (Maley et al., 2019; Sebok et al., 2021). This hypothesis, however, remains debated. Hoekstra et al. (2020) questioned whether heat‐induced improvements in glucose metabolism are directly attributable to intracellular HSPs (iHSPs). Supporting this uncertainty, Pallubinsky et al. (2020) reported reduced FPG and FPI concentrations despite no change in intracellular skeletal muscle HSP‐72. Nevertheless, the heat stimulus employed by Pallubinsky et al. (2020) was relatively mild (∼35°C ambient‐air exposure) and may therefore not have achieved the core temperature required for HSP upregulation. Although the optimal heat dose required to induce HSP upregulation is yet to be determined, Gibson et al. (2016) suggested that maintaining a core temperature above 38.5°C for more than 27 min is necessary to induce HSP mRNA transcription in white blood cells. Consequently, the lack of increased HSP72 in Pallubinsky et al. (2020) does not necessarily exclude a role for iHSPs in mediating heat‐induced improvements in glucose regulation. Similarly, chronic cold exposure has been associated with reduced FPG concentrations and improved glucose regulation (Blondin et al., 2014, 2017; Hanssen et al., 2015; Sellers et al., 2024), although null effects on FPG (Blondin et al., 2017; Lubkowska et al., 2010) and glucose handling have also been reported (Remie et al., 2021). Cold‐induced effects on glucose metabolism have been suggested to be temperature‐dependent, with greater responses observed in more severe cold exposure (Ivanova & Blondin, 2021). This may partly explain the lack of change reported in studies employing milder cooling protocols (Remie et al., 2021).

The clinical relevance of the observed acute reduction in glycaemia remains uncertain, as the significance of a given change in glucose concentration largely depends on baseline glycaemic status and metabolic health. Although based on a single study, the observed reduction in glycaemia suggests that acute contrast exposure may exert physiologically meaningful effects on glucose regulation. The absence of changes in glycaemia and HbA1c following chronic contrast exposure may reflect an insufficient thermal stimulus compared with targeted heat or cold therapy, although this remains uncertain. While the findings are supported by methodologically robust studies with a low risk of bias, the evidence base is limited to two studies, highlighting the need for further research to clarify these effects.

With respect to lipid metabolism, the minor increases reported by Podstawski et al. (2021) have been linked to haemoconcentration due to excessive sweating without fluid replacement. In addition, sauna exposure has been proposed to promote lipolysis and increase circulating FFAs, potentially via catecholamine release and sympathetic nervous system activation (Pilch et al., 2010).

Previous studies of isolated heat and cold exposure suggest that both stimuli can influence lipid metabolism, although findings remain inconsistent. Acute heat exposure has been reported to increase HDL‐C and FFAs following moderate heat stress and reduce TC, TG, and LDL‐C under higher thermal loads (Yamamoto et al., 2003). In contrast, acute cold exposure has been suggested to stimulate lipid metabolism, leading to increases in FFAs and TGs (Blondin et al., 2015; Braunsperger et al., 2025; Sellers et al., 2021). This is consistent with evidence indicating that acute cold exposure enhances both fat and carbohydrate oxidation to meet increased energy demands (Vallerand & Jacobs, 1989).

Repeated sauna exposure has also been proposed to improve lipid profiles through mechanisms resembling moderate‐intensity exercise, including sympathetic activation (Gryka et al., 2014; Pilch et al., 2010). Consistent with this hypothesis, chronic heat exposure is associated with reductions in LDL‐C, HDL‐C and TC (Ely et al., 2019; Pallubinsky et al., 2020), although TG appears unchanged (Imamura et al., 2001; Leppaluoto et al., 1986).

Overall, the available evidence suggests that contrast exposure has limited measurable effects on glucose and lipid concentrations, despite several plausible physiological mechanisms through which heat and cold exposure may influence these outcomes. The acute reduction in glucose concentrations and trends towards altered lipid concentrations suggest that contrast exposure may elicit metabolic responses. However, these findings were reported in a single study, and their clinical relevance remains uncertain, as changes in glucose concentrations largely depend on baseline metabolic status and the observed lipid changes were modest. In contrast, the absence of changes in glycaemia, HbA1c and lipid concentrations following chronic contrast exposure may reflect an insufficient thermal stimulus compared with isolated heat or cold interventions, although this remains speculative. While the acute findings originated from a methodologically robust study judged to be at low risk of bias, the overall evidence base remains limited. This highlights the need for further well‐designed studies to determine whether repeated contrast exposure can produce clinically meaningful metabolic adaptations.

Evidence regarding markers of inflammatory responses to contrast exposure was also limited. Acute contrast exposure consistently increased circulating inflammatory markers, whereas chronic contrast exposure did not produce sustained changes in resting inflammatory status (Table 3). The acute pro‐inflammatory response, however, was reported to be attenuated following chronic contrast exposure (Table 3).

The acute inflammatory response following contrast exposure is broadly consistent with that reported following isolated heat exposure. Acute heat exposure has been shown to increase circulating IL‐6 concentrations, with larger increases occurring following greater elevations in body temperature and longer or more intense heat exposures (Atencio et al., 2025; Behzadi et al., 2020; Gravel et al., 2021). Furthermore, individuals with higher levels of chronic low‐grade inflammation, such as older adults, appear to exhibit greater IL‐6 responses to heat stress (Behzadi et al., 2020). These findings suggest that the increase in IL‐6 observed following contrast exposure likely reflects heat‐induced physiological stress (Pilch et al., 2023).

IL‐6 has been proposed to initiate a subsequent anti‐inflammatory response through the release of cytokines such as IL‐10 (Petersen & Pedersen, 2005). However, no significant changes in IL‐10 concentrations were observed following acute contrast exposure. Similarly, several acute heat exposure studies have reported no detectable increase in circulating IL‐10, although baseline and post‐exposure concentrations were below the assay detection limit, making it difficult to determine whether a biological response occurred (Behzadi et al., 2020; Gravel et al., 2021). It has also been suggested that a minimum IL‐6 response may be required before the downstream anti‐inflammatory cascade is initiated, although this threshold has yet to be established (Shoelson et al., 2006).

The unchanged concentrations of inflammatory markers following chronic contrast exposure are consistent with reports from repeated heat and cold exposure studies, which generally showed unchanged baseline inflammatory markers (Hoekstra et al., 2018; James et al., 2023; Leppaluoto et al., 2008; Pallubinsky et al., 2020; Versteeg et al., 2023). The attenuation of acute pro‐inflammatory responses following chronic contrast exposure may indicate reduced physiological strain during subsequent exposures, consistent with the partial thermophysiological adaptations.

The clinical relevance of these findings depends on the distinction between transient inflammatory responses and chronic low‐grade inflammation. Chronic low‐grade inflammation contributes to insulin resistance, endothelial dysfunction, atherosclerosis, T2D and CVD (Bastard et al., 2006; Ely et al., 2018; Shoelson et al., 2006). Although debated, persistently elevated levels of IL‐6 have been associated with impaired metabolic health, whereas transient increases in IL‐6 have been suggested to enhance glucose uptake and insulin action (Hoekstra et al., 2020). This distinction is why the acute inflammatory response observed following acute contrast exposure should not necessarily be interpreted as detrimental.

In addition to cytokines, HSPs have been proposed as mediators linking thermal exposure with metabolic adaptations. Intracellular HSP72 expression is reduced in individuals with T2D, and animal studies suggest that increasing intracellular HSP72 may improve insulin sensitivity and glucose regulation (Hoekstra et al., 2020). Conversely, extracellular HSPs have generally been associated with systemic inflammation (Hoekstra et al., 2020). Although acute increases in HSP70 were observed following acute contrast exposure, the present evidence is insufficient to determine whether repeated contrast exposure meaningfully influences these pathways.

Overall, the available evidence suggests that contrast exposure elicits transient inflammatory responses characteristic of acute physiological stress without producing sustained changes in resting inflammatory status. Although attenuation of the acute inflammatory response following chronic contrast exposure may indicate partial adaptation, current evidence is insufficient to conclude that contrast exposure improves chronic systemic inflammation or cardiometabolic health. This conclusion is limited by the small evidence base, with chronic inflammatory outcomes reported in only a single study. Future studies should assess a broader panel of inflammatory markers, including C‐reactive protein and tumour necrosis factor‐α, to characterise these effects better.

### 4.3 Potential mechanisms of adaptation

The alternation between heating and cooling creates a physiological stimulus that differs from isolated heat exposure, as it repeatedly challenges both thermoregulatory and cardiovascular control mechanisms. Whereas traditional HA protocols rely on sustained hyperthermia to induce adaptation (Taylor, 2014), contrast therapy requires the body to repeatedly transition between opposing homeostatic responses for heat conservation and heat dissipation. This raises an important question regarding the underlying adaptive stimulus. On one hand, the repeated cooling phases may reduce cumulative heat strain, thereby preventing the thermal stimulus from exceeding the threshold required to elicit the full spectrum of HA adaptations. On the other hand, the continual switching between opposing physiological states may itself constitute a distinct adaptive stimulus that promotes physiological flexibility rather than HA. Whether this repeated thermal alternation confers physiological benefits beyond those achieved through isolated heat or cold exposure remains unknown and represents an important avenue for future research.

### 4.4 Limitations of evidence and review processes

Several methodological limitations should be acknowledged. Study selection, data extraction and quality assessment were conducted by a single reviewer, which may have increased the risk of bias. However, this approach may also have reduced variability in assessment. Three major databases were searched; however, some relevant studies may still have been missed. Non‐English publications without available translations were excluded. This may have led to the omission of potential evidence, particularly from Eastern Europe, where the popularity of sauna bathing has resulted in a substantial body of research.

The limitations of the evidence should also be noted. The number of studies investigating chronic contrast exposure is small and largely originates from a single research group (six out of seven studies). With studies exhibiting overall low methodological study quality, the ability to draw conclusions is limited. Moreover, study protocols vary significantly, and only one trial included a control group without contrast therapy, complicating comparisons between studies and reducing the strength and generalisability of the evidence.

Despite these limitations, this review provides, to our knowledge, the first systematic assessment of the effects of both acute and chronic contrast therapy, synthesising current evidence and identifying key gaps to guide future research.

### 4.5 Implications for practice and research

The current evidence base on the effects of contrast exposure is too limited and inconsistent to support clinical or public health recommendations. Therefore, well‐designed, randomised, controlled intervention studies are essential to assess the acute and, especially, long‐term effects of contrast therapy. Future research should systematically examine vascular and metabolic outcomes with gold‐standard techniques, as these remain notably underexplored compared to parameters such as BP, HR and core temperature. Standardisation of protocols, particularly cooling modality, temperature and exposure duration, is necessary to enhance comparability and identify optimal heating and cooling conditions, exposure times, and treatment frequencies. Longer‐term follow‐up is also required to evaluate the durability of adaptations and whether ongoing exposure is needed.

Finally, while contrast therapy may hold value as a standalone preventive strategy, future studies could also explore its integration with established interventions, such as structured exercise programmes. At present, however, evidence for combined approaches remains limited, and it is unclear whether adding contrast exposure to exercise training provides meaningful additional vascular or haemodynamic benefits beyond exercise alone (Laukkanen & Kunutsor, 2024). Given that both exercise and thermal stimuli independently influence vascular function and haemodynamic load through partly overlapping mechanisms (e.g., endothelial function and autonomic modulation) (Cullen et al., 2020), there is a plausible rationale for investigating whether combined exposure could yield additive or complementary effects.

### 4.6 Conclusion

Evidence regarding the cardiovascular and metabolic effects of contrast therapy remains limited, precluding firm conclusions regarding its overall benefits. Nevertheless, there are indications that contrast therapy may induce partial heat adaptation, although its effects on overall metabolic and vascular health appear minimal. Therefore, well‐designed, high‐quality, controlled studies are essential to clarify its acute and long‐term effects and to determine optimal protocols for potential preventive use.

## AUTHOR CONTRIBUTIONS

V. de Haan: Conceptualisation, writing – original draft, writing‐ review & editing, visualisation. G. Plasqui: Conceptualisation, writing – review & editing, visualisation. H. Pallubinsky: Conceptualisation, supervision, writing – review & editing, visualisation. All authors have read and approved the final version of this manuscript and agree to be accountable for all aspects of the work in ensuring that questions related to the accuracy or integrity of any part of the work are appropriately investigated and resolved. All persons designated as authors qualify for authorship, and all those who qualify for authorship are listed.

## CONFLICT OF INTEREST

None declared.

## GENERATIVE AI STATEMENT

The authors confirm that no artificial intelligence tools, including large language models (LLMs), were used in the drafting or revision of this manuscript. All content was conceived, written, and approved solely by the authors.

## Data Availability

Search strategies are described in the Methods section, with the full strategy provided in Appendix A. All data analysed during this systematic review is included in this published article and its appendices. No primary datasets were generated during the study.

## APPENDIX A

### APPENDIX A.1 SEARCH STRATEGY

**TABLE A1:** Overview of the systematic literature search.

Database	Search date	Search string	Filters	Records
PubMed (MEDLINE)	13 June 2025	(Cryotherapy[MeSH] OR cold exposure OR cold water immersion OR cold therapy OR cryotherapy OR Hot Temperature[MeSH] OR heat exposure OR heat therapy OR hyperthermia OR sauna OR hot water immersion OR thermotherapy) AND (‘cardiovascular function’[tiab] OR ‘endothelial function’[tiab] OR ‘blood pressure’[tiab] OR ‘heart rate variability’[tiab] OR ‘glucose metabolism’[tiab] OR ‘insulin sensitivity’[tiab] OR ‘inflammatory markers’[tiab] OR ‘C‐reactive protein’[tiab] OR ‘lipid profile’[tiab] OR triglycerides[tiab] OR cholesterol[tiab]) AND (passive[tiab] OR therapy[tiab] OR treatment[tiab]) AND (Humans[MeSH] AND (adult[MeSH] OR adult[tiab])) AND english[lang] NOT (review[pt] OR ‘systematic review’[tiab] OR meta‐analysis[pt] OR observational[tiab] OR cohort[tiab] OR case‐control[tiab])	Language: English Population: adult humans Excluded: reviews, observational studies, meta‐analyses, cohort and case–control studies	764
Scopus	17 June 2025	(TITLE‐ABS‐KEY(cryotherapy OR ‘cold exposure’ OR ‘cold water immersion’ OR ‘cold therapy’ OR ‘heat exposure’ OR ‘heat therapy’ OR hyperthermia OR sauna OR ‘hot water immersion’ OR thermotherapy)) AND (TITLE‐ABS‐KEY(‘cardiovascular’ OR ‘endothelial function’ OR ‘blood pressure’ OR ‘heart rate variability’ OR ‘glucose metabolism’ OR ‘insulin sensitivity’ OR ‘inflammation’ OR ‘C‐reactive protein’ OR ‘lipid profile’ OR triglycerides OR cholesterol)) AND (TITLE‐ABS‐KEY(passive OR therapy OR treatment)) AND (LANGUAGE(english)) AND (NOT DOCTYPE(re) AND NOT TITLE‐ABS‐KEY(‘systematic review’ OR ‘meta‐analysis’ OR observational OR cohort OR ‘case‐control’))	Language: English Population: adult humans Excluded: reviews, meta‐analyses, observational studies, cohort, case–control studies	664
Web of Science	17 June 2025	TS = (cryotherapy OR ‘cold exposure’ OR ‘cold water immersion’ OR ‘cold therapy’ OR ‘heat exposure’ OR ‘heat therapy’ OR hyperthermia OR sauna OR ‘hot water immersion’ OR thermotherapy) AND TS = (‘cardiovascular function’ OR ‘endothelial function’ OR ‘blood pressure’ OR ‘heart rate variability’ OR ‘glucose metabolism’ OR ‘insulin sensitivity’ OR ‘inflammatory markers’ OR ‘C‐reactive protein’ OR ‘lipid profile’ OR triglycerides OR cholesterol) AND TS = (passive OR therapy OR treatment) AND TS = (adult) NOT TS = (‘systematic review’ OR ‘meta‐analysis’ OR review OR observational OR cohort OR ‘case‐control’)	Language: English Excluded: reviews, meta‐analyses, observational, cohort, or case–control studies	105

## APPENDIX B

### APPENDIX B.1 CALCULATIONS

For the within‐subject pre–post comparison, the following formula was employed to estimate Cohen's *d* (Lenhard & Lenhard, 2022):

$$d=\frac{M_{\mathrm{diff}}}{\sigma \sqrt{2\left(1-\rho \right)}}$$

The denominator represents the standard deviation of the difference between the pre‐ and post‐score (adjusted for correlation), σ is the pooled standard deviation of pre‐ and post‐scores, and ρ is the correlation between them, assumed to be 0.5 in the absence of reported values.

For the comparison between the pre‐post values from the first and the last exposure (difference‐in‐differences), the difference in change scores between the first (*D*_1_) and last (*D*_2_) exposure was divided by the pooled standard deviation (SD_1_; SD_2_) of those change scores, with *r* representing the correlation between the difference scores, in accordance with general guidelines for effect size estimation in repeated‐measures designs (Lakens, 2013; Morris & DeShon, 2002):

$$d=\frac{D_{1}-D_{2}}{\sqrt{\frac{SD_{1}^{2}+SD_{2}^{2}-2rSD_{1}SD_{2}}{2}}}$$

For DiD analyses, change scores were calculated from the reported means. Standard deviations of the change scores were estimated from the reported standard deviations, assuming a pre‐post correlation of r = 0.5, as no correlation coefficients were reported (Appendix B).

$$SD_{diff}=\sqrt{SD_{pre}^{2}+SD_{post}^{2}-2rSD_{pre}SD_{post}}$$
